# Human neutrophil development and functionality are enabled in a humanized mouse model

**DOI:** 10.1073/pnas.2121077119

**Published:** 2022-10-21

**Authors:** Yunjiang Zheng, Esen Sefik, John Astle, Kutay Karatepe, Hasan H. Öz, Angel G. Solis, Ruaidhrí Jackson, Hongbo R. Luo, Emanuela M. Bruscia, Stephanie Halene, Liang Shan, Richard A. Flavell

**Affiliations:** ^a^Department of Immunobiology, Yale University, New Haven, CT 06520;; ^b^Department of Pathology, Medical College of Wisconsin, Milwaukee, WI 53226;; ^c^Department of Cell Biology, Yale University, New Haven, CT 06520;; ^d^Section of Pediatric Pulmonology, Allergy, Immunology & Sleep Medicine, Department of Pediatrics, Yale University School of Medicine, New Haven, CT 06520;; ^e^Department of Microbiology, University of Pennsylvania, Philadelphia, PA 19104;; ^f^Department of Immunology, Harvard Medical School, Boston, MA 02115;; ^g^Department of Laboratory Medicine, The Stem Cell Program, Boston Children’s Hospital, Boston, MA 02115;; ^h^Department of Pathology, Harvard Medical School, Boston, MA 02115;; ^i^Section of Hematology, Department of Internal Medicine, Yale Comprehensive Cancer Center, Yale University School of Medicine, New Haven, CT 06520;; ^j^Division of Infectious Diseases, Department of Medicine, Washington University School of Medicine in St. Louis, St. Louis, MO 63110;; ^k^Howard Hughes Medical Institute (HHMI), New Haven, CT 06520;; ^l^Yale Stem Cell Center, Yale University, New Haven, CT 06520;; ^m^Department of Pathology, Yale University School of Medicine, New Haven, CT 06520

**Keywords:** neutrophils, innate immunity, bacterial infection, humanized mouse

## Abstract

Mice with a functional human immune system serve as an invaluable tool to study the development and function of the human immune system in vivo. A major technological limitation of all current humanized mouse models is the lack of mature and functional human neutrophils in circulation and tissues. To overcome this, we generated a humanized mouse model named MISTRGGR, in which the mouse granulocyte colony-stimulating factor (G-CSF) was replaced with human G-CSF and the mouse G-CSF receptor gene was deleted in existing MISTRG mice. By targeting the G-CSF cytokine-receptor axis, we dramatically improved the reconstitution of mature circulating and tissue-infiltrating human neutrophils in MISTRGGR mice. Moreover, these functional human neutrophils in MISTRGGR are recruited upon inflammatory and infectious challenges and help reduce bacterial burden. MISTRGGR mice represent a unique mouse model that finally permits the study of human neutrophils in health and disease.

## Significance

A major limitation of all current humanized mouse models is lack of mature human neutrophils in circulation and tissues. To overcome this, we targeted the granulocyte colony-stimulating factor (G-CSF) cytokine-receptor axis and generated a humanized mouse model named MISTRGGR, in which the cytokine G-CSF was humanized (MISTRGG) and the murine G-CSF receptor was ablated (MISTRGGR). The reconstitution of mature circulating and tissue-infiltrating human neutrophils was dramatically improved in MISTRGGR mice. Human neutrophils generated in MISTRGGR mice are functional and respond robustly to inflammatory and infectious stimuli. MISTRGGR mice represent a unique, highly influential approach that for permits the study of human neutrophils in health and disease and may enable applications in unexplored and wider areas of translational research.

Mice serve as valuable tools for the study of in vivo immune responses. Although the overall structure and composition of the murine and human immune systems are comparable at many levels, extensive evolutionary pressures over millions of years have resulted in significant changes (1–4). Differences between the human and murine immune systems have been widely characterized in blood. Transcriptional analysis of mouse and human cells show similarities in lineage-defining genes and aspects of cell identity, yet many human and mouse immune cells show striking differences in their activities and effector or regulatory mechanisms (5). Neutrophils are one such cell type for which human or murine specific effects drive differences in immunity and pathogenesis.

Neutrophils are indispensable during injury, infection, and as more recently appreciated, in homeostatic immune surveillance in both humans and mice (6–9). They are the most abundant circulating leukocytes and play essential roles in fending against microbial infections through a variety of mechanisms, including phagocytosis, release of reactive oxygen species (ROS), antimicrobial peptides and proteases, and by their unique formation of neutrophil extracellular traps (NETs) (9, 10). Both human and mouse neutrophils produce various cytokines and chemokines to influence inflammation and regulate adaptive immune responses (11). Yet, there are significant differences in the maturation, migration, and function of human and mouse neutrophils. The structure of key neutrophil molecules (selectins, FcαRI, serine proteases) (12–15), expression of cytokines (interleukin [IL]-10, IL-17) (16, 17), activation pathways of ROS (18, 19), and cytotoxic granular content (defensins, bactericidal enzymes) (14, 20–22) display major cross-species alterations that can affect migration, signaling, effector mechanisms, and neutrophil activation (23). These human-to-mouse differences in the overall biology of neutrophils have striking implications for immunity against infections, the pathogenesis of cancer, and health (4, 6–23).

Neutrophils are pivotal to the human innate defense against various bacterial and fungal pathogens, including *Listeria monocytogenes*, *Klebsiella pneumoniae*, *Pseudomonas aeruginosa*, and *Candida auris* (24–27). In contrast to mice, for which neutrophil deficiencies do not cause a progressive health deterioration under specific pathogen-free conditions (28), neutropenic humans suffer greatly from exposure to microbials in daily life (29), suggesting an even more crucial role of neutrophils in immune defense against infections in humans. Individuals with severe congenital neutropenia, as well as patients undergoing chemotherapy and bone marrow transplantation, are commonly treated with recombinant human granulocyte colony-stimulating factor (G-CSF) (filgrastim). G-CSF drives granulopoiesis and improves and accelerates the recovery of circulating neutrophil counts, thus reducing the frequency and severity of infections (30, 31).

In various human and murine tumors, neutrophils compose a significant part of tumor-infiltrating myeloid cells and can display both pro- and antitumor effects (23, 32, 33). Although recent work has provided clues to how neutrophils play a complex role in pathologies, such as cancer progression, significant gaps remain in understanding the regulation of neutrophil infiltration and survival within the tumor microenvironment and the switch between pro- and antitumor phenotypes.

More recently, there has been a growing appreciation for the role of neutrophils in homeostatic immune surveillance, as well as tissue physiology in both humans and mice. Neutrophils can infiltrate multiple tissues, like the spleen and lung, even at the steady state and participate in local immune homeostasis (6–8, 34). Yet, it remains unknown to what extent infiltrating neutrophils contribute to normal tissue physiology and immune surveillance, especially in humans, as approaches to study and manipulate human neutrophils in human tissues are limited.

Conflicting reports in mouse studies and a general lack of data about the function of human neutrophils have created a fundamental need for the development of humanized mice that allow for the development and maturation of functional human neutrophils, which are capable of responding to inflammation. Overall, there is an urgent need for models that support in vivo studies of human neutrophils as murine findings have failed to translate to humans.

The development of humanized hemato-lymphoid system mice (referred to here as “humanized” mice) that are generated by transplantation of human hematopoietic cells into various strains of immune-compromised mice has provided a leap forward for studying human immune function in vivo. MISTRG mice—which harbor human cytokines MCSF, IL-3/GM-CSF, THPO, and human SIRPα—have highly improved maturation and function of human monocytes, macrophages, natural killer cells, as well as the general humanization of the immune system (35). Strikingly, however, no system that supports circulating human neutrophils currently exists. Here, we describe a innovative humanized mouse model by the humanization of the cytokine G-CSF and ablation of the murine G-CSF receptor (G-CSFR), to finally allow human neutrophil development and survival in an in vivo model system. With successful human neutrophil reconstitution in the periphery, this humanized mouse model can now be applied to study human neutrophils and unravel their major contribution to health and disease.

## Results

### Generation of Human G-CSF Knockin Mice (MISTRGG).

G-CSF is the major cytokine that regulates the commitment, development, maturation, and survival of neutrophilic granulocytes (36–39). Deletion of the murine *G-CSF* gene revealed physiologic roles for this cytokine in granulopoiesis at both steady state and conditions of inflammation and infections (37). G-CSF is produced by a variety of cells, including macrophages, T cells, endothelial cells, and fibroblasts upon inflammation signals, and in turn induces proliferation of neutrophilic progenitors and the release of postmitotic neutrophils from the bone marrow (40, 41). Human and murine G-CSF proteins display a relatively high degree of amino acid sequence homology (73%) (42). Early studies have shown receptor-binding cross-reactivity between human and mouse G-CSF (43); however, little is known of the efficacy of these proteins in a cross-species bone marrow microenvironment.

**Fig. 1.**
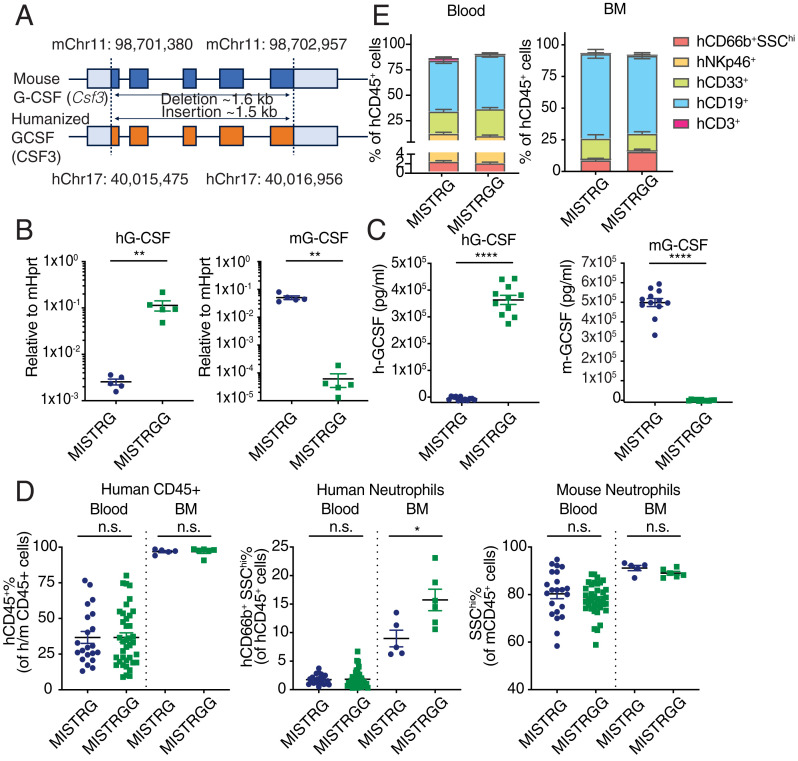
G-CSF humanization enhances neutrophil commitment in the bone marrow (BM). (*A*) Schematic design of human G-CSF knockin replacement. The entire mouse ORF (in blue) is replaced by human ORF (in orange). Both 5′ and 3′ UTR sequences are primarily murine. (*B*) Relative expression of human and mouse G-CSF mRNA in the bone marrow at 3 h after LPS administration (50 μg, intraperitoneally; *n* = 5 mice). (*C*) Human and mouse G-CSF proteins measured in plasma at 3 h after LPS administration (50 μg, intraperitoneally) (MISTRG, *n* = 12; MISTRGG, *n* = 11 mice). Data pooled from two independent experiments. (*D*) Frequencies of human hematopoietic cells (hCD45^+^), human neutrophils (hCD66b^+^ SSChi), and mouse neutrophils (mCD45^+^ SSChi) in the blood (MISTRG, *n* = 21; MISTRGG, *n* = 38 mice) and bone marrow (MISTRG, *n* = 5; MISTRGG, *n* = 6 mice). (*E*) Frequencies of human lineages in the blood (MISTRG, *n* = 14; MISTRGG, *n* = 20 mice) and the bone marrow (MISTRG, *n* = 5; MISTRGG, *n* = 6 mice) at 7 wk postengraftment. (*D* and *E*) Data pooled from at least three independent experiments. Mice were irradiated with 150 Rads and intrahepatically injected with 20,000 human CD34^+^ cells at 1 to 3 d after birth. (*B*–*E*) Data are shown as mean ± SEM. *P* values determined by two-tailed Mann–Whitney test (*P < 0.05; **P < 0.01; ***P < 0.001; ****P < 0.0001). Each dot represents an individual mouse.

Given the crucial role of G-CSF in regulating granulopoiesis, we hypothesized that the humanization of cytokine G-CSF will favor human neutrophil reconstitution in humanized MISTRG mice. Thus, we generated mice that are deficient in mouse *G-CSF* (*Csf3*) and express human *G-CSF* (*CSF3*), instead using a CRISPR/Cas9 system (44), referred to as MISTRGG (G for G-CSF^hh^) mice hereafter. The construct was designed to replace the entire mouse *G-CSF* open reading frame (ORF; ∼1.6 kb) with the human ORF, but to maintain the promotor, 5′ and 3′ UTRs primarily of mouse origin (Fig. 1*A*). We confirmed the loss of mouse G-CSF and expression of the human G-CSF in the bone marrow and blood plasma after lipopolysaccharide (LPS) stimulation (Fig. 1 *B* and *C*), a widely accepted protocol that induces systemic G-CSF expression in various tissues and cell types (40, 45). We also confirmed the respective expression of human and mouse G-CSF protein in tissues such as the bone marrow and lung (*SI Appendix*, Fig. S1*A*), and established that, as expected, endothelial cells (40) in liver and lung rapidly produced human G-CSF in levels comparable to mouse G-CSF following LPS stimulation (*SI Appendix*, Fig. S1*B*). Epithelial cells also responded to LPS stimulation by producing G-CSF but to a lesser extent than endothelial cells (*SI Appendix*, Fig. S1*C*).

To assess the effect of G-CSF humanization on granulocyte reconstitution in humanized mice, newborn MISTRGG and control MISTRG mice were intrahepatically injected with human fetal CD34^+^ hematopoietic stem and progenitor cells. As a readout of engraftment, human and mouse immune cells were quantified at 7 to 8 wk postinjection (*SI Appendix*, Fig. S1*D*). Replacement of the mouse *G-CSF* with the human *G-CSF* yielded increased human neutrophil frequencies in the bone marrow at steady state but did not impact circulating neutrophils in blood (Fig. 1*D*). Other major immune human populations—such as T, NK, B, and CD33^+^ myeloid cells—remained unaffected in MISTRGG mice (Fig. 1*E*). The levels of murine neutrophils in circulation and bone marrow were not affected (Fig. 1*D*), which we hypothesize is a result of the functionality of human G-CSF on the mouse G-CSFR due to the reported capability of recombinant human G-CSF to induce murine granulocytic colony formation (43, 46). Thus, expression of the human G-CSF cytokine alone does not significantly enhance neutrophil circulatory levels, which emphasizes the necessity to introduce modifications that further curtail the competition from murine progenitors.

### Murine G-CSFR Deletion Improves Human Neutrophil Reconstitution in Circulation.

**Fig. 2.**
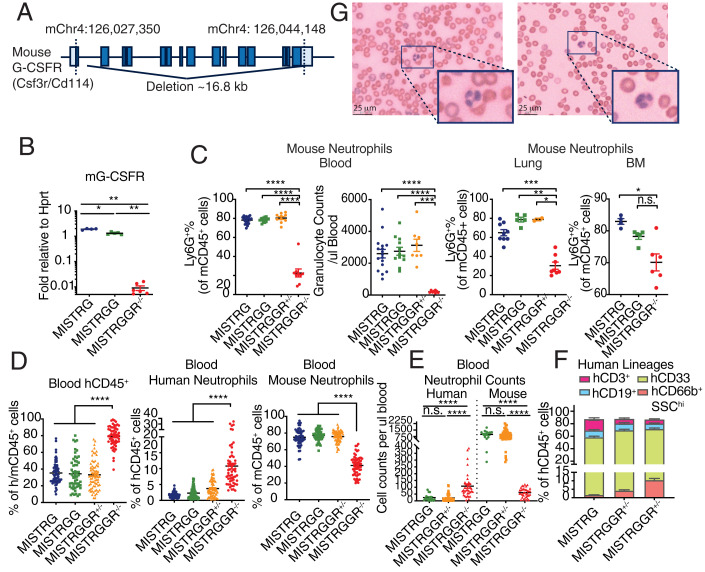
Depletion of murine neutrophils by G-CSFR ablation improves engraftment of human neutrophils in circulation. (*A*) Schematic design of mouse G-CSFR deletion. The entire G-CSFR ORF and first ∼300 bp of 3′ UTR are deleted. (*B*) Relative expression of G-CSFR in bone marrow of MISTRG (*n* = 4), MISTRGG (*n* = 5), MISTRGGR^−/−^ (*n* = 6) mice. Hprt was used as a housekeeping gene. (*C*) Frequencies and numbers of mouse granulocytes (Ly6G^+^) in blood (*Left*), bone marrow (*Right*), and lung (*Center*) of MISTRG (*n* = 17 [blood], *n* = 4 [BM], *n* = 9 [lung]), MISTRGG (*n* = 12 [blood], *n* = 5 [BM], *n* = 5 [lung]), MISTRGGR^+/−^ (*n* = 8 [blood], *n* = 2 [lung]) and MISTRGGR^−/−^ (*n* = 8 [blood], *n* = 6 [BM], *n* = 8 [lung]) mice. Data were representative of at least three independent experiments. Whole lung tissues were analyzed without perfusion. (*D*–*F*) Characterization of human engraftment in the blood of MISTRG, MISTRGG, MISTRGGR^+/−^ and MISTRGGR^−/−^ mice at 7 wk postengraftment. Data pooled from at least eight independent experiments. Mice were irradiated with 150 Rads and intrahepatically injected with 15,000∼30,000 human fetal liver CD34^+^ cells at 1 to 3 d after birth. (*D*) Frequencies of human hematopoietic cells (hCD45^+^), human neutrophils (hCD66b^+^ SSChi), and mouse neutrophils (Ly6G^+^) (MISTRG, *n* = 58; MISTRGG, *n* = 54; MISTRGGR^+/−^, *n* = 72; MISTRGGR^−/−^, *n* = 58). (*E*) Numbers of human and mouse neutrophils (MISTRGG, *n* = 15; MISTRGGR^+/−^, *n* = 53; MISTRGGR^−/−^, *n* = 35). (*F*) Frequencies of human immune lineages (MISTRG, *n* = 58; MISTRGGR^+/−^, *n* = 38; MISTRGGR^−/−^, *n* = 48). (*G*) MGG staining of engrafted MISTRGGR^−/−^ blood smears. Neutrophils from two representative mice are shown in the enlarged box. (*B*–*F*) Data are shown as mean ± SEM. *P* values determined by two-tailed Mann–Whitney test (*P < 0.05; **P < 0.01; ***P < 0.001; ****P < 0.0001). Each dot represents an individual mouse.

Therefore, in order to reduce competition between human and murine granulocytic progenitors over niche space, human G-CSF, and other cytokines and growth factors, we deleted the mouse G-CSF receptor (*G-CSFR*, also known as *Csf3r/Cd114*) in MISTRGG mice, referred to as MISTRGGR (R for *G-CSFR*^−/−^) mice hereafter, as G-CSFR deficiency is expected to cause an 80% decrease in mouse neutrophils in circulation as well as the bone marrow (47). We designed CRISPR guides targeting both the start codon and at the beginning of 3′ UTR, resulting in a deletion of ∼16.8 kb spanning the entire mouse ORF (Fig. 2*A*). The loss of G-CSFR was confirmed at transcript level (Fig. 2*B*). To assess the outcome of G-CSFR deletion on mouse neutrophils, we quantified mouse neutrophils by flow cytometry in MISTRGGR^−/−^ mice. As anticipated, MISTRGGR^−/−^ mice had a significant reduction of mouse neutrophils in the blood, bone marrow, and peripheral tissues, such as lungs (Fig. 2*C*).

Given the reduced number of mouse neutrophils in MISTRGGR^−/−^, we wanted to test whether circulating and peripheral human neutrophil proportions and numbers were affected in engrafted mice. Newborn MISTRGGR^−/−^ and control littermates were again injected with fetal CD34^+^ cells, and human engraftment were assessed similarly as in Fig. 1. Remarkably, overall humanization quantified by human CD45^+^ expression was enhanced in MISTRGGR^−/−^ compared with MISTRGGR^+/−^, MISTRGG, and MISTRG groups. This enhanced humanization corresponded to a marked increase in human neutrophils (CD66b^+^ SSC^hi^) as well as an accompanied reduction in mouse neutrophils (Ly6G^+^), particularly in blood but also in spleens and livers (Fig. 2 *D* and *E* and *SI Appendix*, Fig. S2 *A* and *B*). The reconstitution of other major immune human populations, such as B cells and CD33^+^ myeloid cells, was unaffected (Fig. 2*F* and *SI Appendix*, Fig. S2*C*). A definitive identification of human neutrophils was achieved by May-Grunwald Giemsa (MGG) staining in blood smears (Fig. 2*G*). These cells (as seen in the enlarged boxes in Fig. 2*G*) had segmented nuclei with approximately three lobes and a light pink cytoplasm, which is in accordance with the distinct morphology of mature human neutrophils (48).

There’s been growing appreciation for the phenotypic heterogeneity that may reflect different cellular states of neutrophils with varying maturation or activation statuses (49). Neutrophils freshly released from the bone marrow express high levels of L-selectin (CD62L), CXCR2, and low levels of CXCR4. In circulation, they can “age” by substantial down-regulation of CD62L and CXCR2 expression, and conversely up-regulation of CXCR4 expression, which is required for homing back to the bone marrow for macrophage-mediated clearance (50). In our model, both “fresh” (CD62L^hi^ CXCR2^hi^ CXCR4^lo^) and “aged” (CD62L^lo^ CXCR2^lo^ CXCR4^hi^) neutrophils were observed from the blood of engrafted MISTRGGR^−/−^ mice (*SI Appendix*, Fig. S2*E*). Fresh neutrophils take up about 80 to 90% of total circulating human neutrophils at week 7 postengraftment and 70 to 85% at week 10 postengraftment (*SI Appendix*, Fig. S2 *D* and *E*). Overall, the presence of phenotypically distinct human neutrophil populations has suggested that neutrophil homeostasis is properly maintained in MISTRGGR mice.

### Reconstituted Human Neutrophils Are Capable of Phagocytosis, ROS Production, NET Formation, and Chemotaxis.

**Fig. 3.**
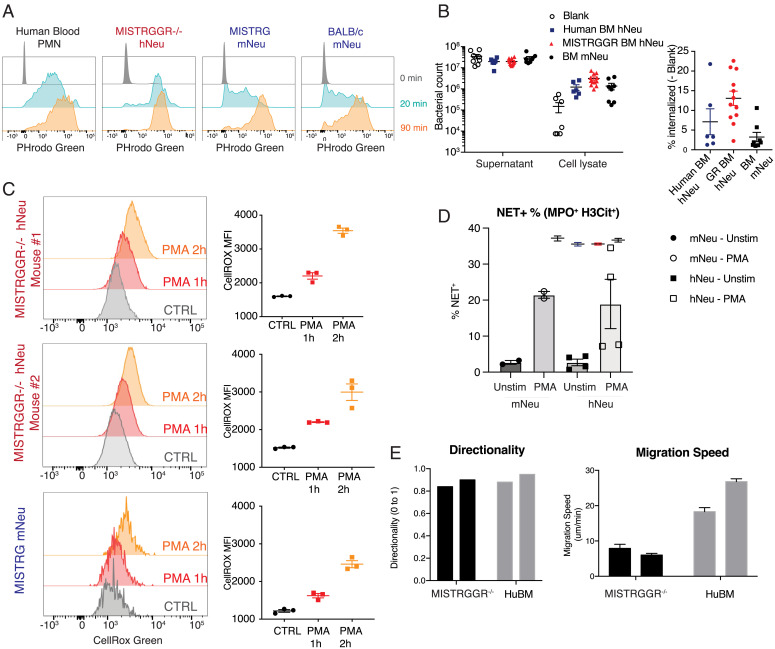
Functionalities of reconstituted human neutrophils in MISTRGGR. (*A*) Representative flow cytometry analysis of phagocytosis by human blood polymorphonuclear leukocytes (PMNs) (*Left*), reconstituted human neutrophils from the blood of engrafted MISTRGGR^−/−^ (*Center Left*), mouse neutrophils from the blood of engrafted MISTRG (*Center Right*), and unengrafted BALB/c (*Right*). Blood samples were incubated with pHrodo Green *E. coli* BioParticles Conjugate (10 µg per 50 µL blood) for the indicated times and fluorescent signals were analyzed by flow cytometry. (*Top*) 0 min; (*Middle*) 20 min; (*Bottom*) 90 min. Data are representative of at least two independent experiments. (*B*) Phagocytosis of *P. aeruginosa* by human and mouse neutrophils in vitro. Equal numbers of isolated human and mouse neutrophils (100,000 cells per well) from bone marrow of humanized MISTRGGR^−/−^ and human neutrophils from human healthy donors were cultured with live *P. aeruginosa* at a multiplicity of infection of 1:10 in 37 °C for 30 min. Bacterial counts (*Left*) and frequencies of internalized bacteria (*Right*) were measured from culture supernatant and cell lysates separately. (*C*) Human neutrophils isolated from the bone marrow of MISTRGGR were stimulated with PMA (10 ng/mL) for 1 or 2 h at 37 °C and stained with CellROX Green. ROS production was analyzed by flow cytometry and MFI was calculated. (*D*) Quantification of NETs-forming neutrophils. Isolated human and mouse neutrophils from engrafted bone marrow were cultured unstimulated or with 20 nM PMA for 4 h at 37 °C. MPO and histone H3 were detected by flow cytometry. Each point represents cells isolated from one individual mouse. Data were pooled from two independent experiments. (*E*) Ex vivo Chemotactic ability of human neutrophils isolated from engrafted MISTRGGR mice and fresh human bone marrow control were compared using the EZ-taxiscan chamber. Directionality and speed of human neutrophils were measured as they migrated toward IL-8 (1 μM).

We further tested the capacity of reconstituted human neutrophils to phagocytose inflammatory targets in an in vitro assay using fluorogenic *Escherichia coli* bioparticles (51). Human neutrophils from MISTRGGR^−/−^ blood were capable of phagocytosis with efficacy similar to polymorphonuclear leukocytes from human blood (Fig. 3*A*). These observations were specific to human and mouse neutrophils and were not observed in other lineages capable of phagocytosis such as human B cells (Fig. 3*A* and *SI Appendix*, Fig. S3*A*). In addition, in an in vitro phagocytosis assay where human neutrophils isolated from engrafted MISTRGGR^−/−^ bone marrow were incubated with *P. aeruginosa* (PA14), human neutrophils from MISTRGGR mice have exhibited a comparable and even higher rates of phagocytosis than control murine and human donor neutrophils (Fig. 3*B*). The production of ROS by NADPH oxidase upon activation is another crucial effector function of neutrophils. Therefore, we measured ROS production by human and mouse neutrophils isolated from engrafted MISTRGGR and MISTRG mice after stimulation with phorbol 12-myristate 13-acetate (PMA) alone or a combination of tumor necrosis factor-α (TNF-α) and fMLP. Both human neutrophils from the bone marrow and blood of MISTRGGR exhibited strong ROS production in response to PMA stimulation (Fig. 3*C* and *SI Appendix*, Fig. S3*B*). Blood neutrophils also respond to combination signals of TNF-α and fMLP (*SI Appendix*, Fig. S3*B*). Formation of NETs (9, 10) is another unique feature of neutrophils that greatly impacts their function. We measured the ability of human neutrophils from MISTRGGR mice to undergo NETosis upon PMA stimulation ex vivo. Human neutrophils, like their mouse counterparts, effectively formed NETs measured by flow cytometric evaluation of Myeloperoxidase (MPO) and histone H3 (Fig. 3*D* and *SI Appendix*, Fig. S3*D*).

As a further characteristic of reconstituted human neutrophils, we assessed their chemotactic potential and ability. First, we compared the expression of chemokine receptors on human neutrophils in blood, spleen, and lung. Chemokine receptor expression has been shown to be largely absent or marginal in circulating neutrophils while lung-infiltrating neutrophils up-regulate expression of CCR2, CCR5, CXCR4, CXCR3, CXCR1 and acquire chemokine responsiveness (9, 52). Indeed, reconstituted human neutrophils acquired higher expression of these chemokine receptors in both lung vasculature and interstitium (*SI Appendix*, Fig. S3*C*). To test the chemotactic ability of human neutrophils, we compared the directionality and speed of human neutrophils as they migrated toward IL-8 ex vivo using the EZ-taxiscan chamber. The humanized neutrophils were indeed capable of chemotaxis when compared to purified human neutrophils **(***SI Appendix*, Fig. S3*E*). The directionality of reconstituted human neutrophils from MISTRGGR^−/−^ mice was comparable to human neutrophils from healthy donors whereas the migration of humanized human neutrophils was somewhat slower (Fig. 3*E*, *SI Appendix*, Fig. S3*E*, and Movies S1–S4). Overall, these findings support that human neutrophils from reconstituted mice have phagocytic and chemotactic abilities and respond to chemotactic stimuli as expected.

### Enhanced Bone Marrow Granulopoiesis in MISTRGGR.

**Fig. 4.**
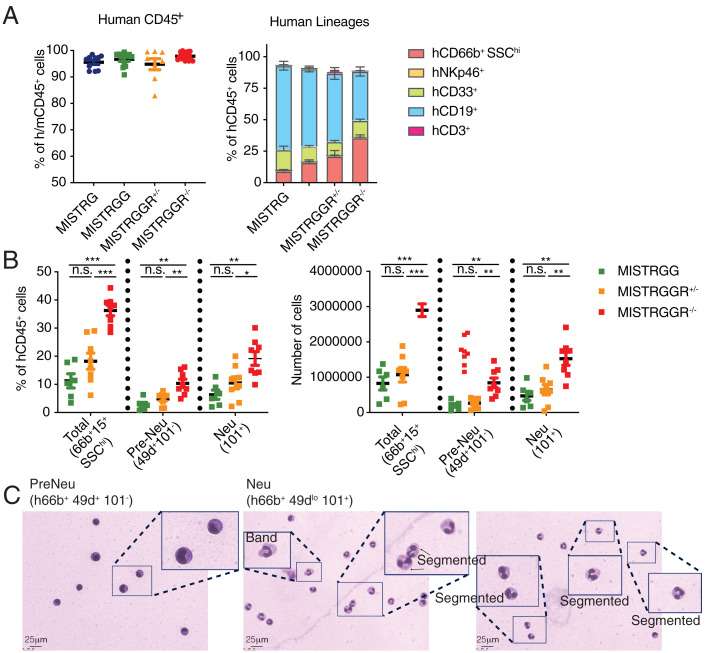
Enhanced bone marrow granulopoiesis in MISTRGGR mice. Bone marrow cells were analyzed at 8 wk postengraftment. (*A*) Frequencies of human hematopoietic cells (hCD45^+^) (*Left*) (MISTRG, *n* = 10; MISTRGG, *n* = 12; MISTRGGR^+/−^, *n* = 8; MISTRGGR^−/−^, *n* = 15 mice), human lineages (*Right*) (MISTRG, *n* = 5; MISTRGG, *n* = 12; MISTRGGR^+/−^, *n* = 8; MISTRGGR^−/−^, *n* = 8 mice). Data pooled from three independent experiments. (*B*) Frequencies (*Left*) and numbers (*Right*) of human neutrophils (hCD66b^+^ SSChi), Pre-Neu (hCD49d^+^ CD101^−^), and Neu (hCD101^+^) in the bone marrow of MISTRGG (*n* = 6 mice), MISTRGGR^+/−^ (*n* = 8 mice), and MISTRGGR^−/−^ (*n* = 8 mice). Data pooled from at least two independent experiments. (*C*) MGG staining of sorted bone marrow Pre-Neu (*Left*) and Neu (*Center* and *Right*) cells. Enlarged boxes highlight various stages of human neutrophil development. (*A* and *B*) Data are shown as mean ± SEM. *P* values determined by two-tailed Mann–Whitney test (*P < 0.05; **P < 0.01; ***P < 0.001). Each dot represents an individual mouse.

One explanation for enhanced circulating neutrophils was enhanced neutrophil development in the bone marrow. While the overall human engraftment (hCD45^+^) in the bone marrow was comparable across different genotypes, MISTRGGR^−/−^ mice had significantly more human neutrophils (CD66b^+^ CD15^+^ SSC^hi^) (Fig. 4 *A* and *B* and *SI Appendix*, Fig. S4*A*). This population of neutrophils and neutrophilic progenitors has been shown to be further separated into CD49d^+^ 101^−^ pre-Neutrophils (Pre-Neu), primarily myelocytes and metamyelocytes, and CD101^+^ Neutrophils (Neu), consisting of band and segmented neutrophils (53). We’ve performed a similar flow cytometric analysis and revealed that G-CSFR ablation improved reconstitution of both pre-Neu and Neu subsets (Fig. 4*B* and *SI Appendix*, Fig. S4*B*), indicating an enhancement throughout all different stages of human neutrophilic granulopoiesis. This universal increase in neutrophil commitment is as expected, given that both earlier proliferation and terminal differentiation of neutrophil progenitors were shown to be induced by G-CSF signaling (54). The developmental stages of neutrophils in pre-Neu and Neu subsets were also confirmed with MGG staining (Fig. 4*C*).

### Functional Human Neutrophils Are Recruited to the Lung upon LPS-Induced Inflammation.

**Fig. 5.**
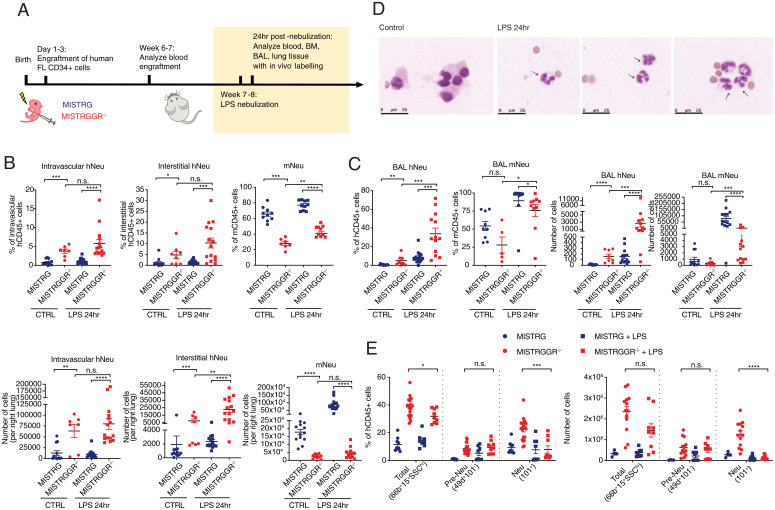
Robust human neutrophil recruitment to the lung upon inflammatory stimuli in MISTRGGR mice. (*A*) Experimental timeline of LPS nebulization. Seven- to 8-wk-old engrafted mice were treated with LPS nebulization (12.5 mg administered over 15 min). BALF and lung tissues were harvested at 24 h after nebulization. Prior to being killed, mice were injected with anti-human CD45 antibody intravenously to label circulating human cells. Interstitial neutrophils and intravascular neutrophils were distinguished by gating on in vivo versus in vitro hCD45 fluorochrome signals. (*B*) Quantifications of intravascular and interstitial human neutrophils (hCD66b^+^ SSChi) (CTRL MISTRG: *n* = 12; CTRL MISTRGGR^−/−^, *n* = 7; LPS MISTRG, *n* = 14; LPS MISTRGGR^−/−^, *n* = 15 mice) and mouse neutrophils (Ly6G^+^) (CTRL MISTRG: *n* = 10; CTRL MISTRGGR^−/−^, *n* = 7; LPS MISTRG, *n* = 10; LPS MISTRGGR^−/−^, *n* = 10 mice) in lungs of MISTRG and MISTRGGR^−/−^ mice at control steady state or 24 h after LPS nebulization. Data pooled from at least three independent experiments. (*C*) Frequencies, and numbers of human neutrophils (CTRL MISTRG: *n* = 12; CTRL MISTRGGR^−/−^, *n* = 8; LPS MISTRG, *n* = 14; LPS MISTRGGR^−/−^, *n* = 13 mice) and mouse neutrophils (CTRL MISTRG: *n* = 9; CTRL MISTRGGR^−/−^, *n* = 5; LPS MISTRG, *n* = 10; LPS MISTRGGR^−/−^, *n* = 10 mice) in BAL at control steady state or 24 h after LPS nebulization. Data pooled from at least three independent experiments. (*D*) MGG staining of cells isolated from BAL at 24 h after LPS nebulization. Arrows: human neutrophils. (*E*) Frequencies (*Top*) (CTRL MISTRG: *n* = 9; CTRL MISTRGGR^−/−^, *n* = 20; LPS MISTRG, *n* = 8; LPS MISTRGGR^−/−^, *n* = 9 mice) and numbers (*Bottom*) (CTRL MISTRG: *n* = 4; CTRL MISTRGGR^−/−^, *n* = 13; LPS MISTRG, *n* = 8; LPS MISTRGGR^−/−^, *n* = 9 mice) of human neutrophils (hCD66b^+^ SSChi), preNeu (hCD49d^+^ CD101^−^), and Neu (hCD101^+^) in the bone marrow of engrafted mice at steady state or 24 h after LPS nebulization. Data pooled from at least two independent experiments. (*B*–*D* and *F*) Data are shown as mean ± SEM. *P* values determined by two-tailed Mann–Whitney test (*P < 0.05; **P < 0.01; ***P < 0.001; ****P < 0.0001). Each dot represents an individual mouse.

As a further characteristic of reconstituted human neutrophils, we assessed their migration and extravasation into peripheral tissues. Analysis of human immune cell composition in engrafted mice has shown a trend of increased human neutrophil infiltration into the spleen, liver, and lung at steady-state in MISTRGGR^−/−^ mice as compared to MISTRG (*SI Appendix*, Fig. S2*B*). Upon septic or aseptic injury, circulating neutrophils exit the bloodstream and extravasate in large amounts into compromised tissues, where they can employ their effector machinery to combat pathogens. LPS nebulization provides an ideal tool to study the recruitment and function of neutrophils in the lung. In this model, mice are exposed to aerosolized LPS, which initiates recruitment of neutrophils into the pulmonary vasculature, transendothelial migration into the lung interstitium, and subsequently transepithelial migration into the alveolar space (55). Neutrophil accumulation in lung vasculature peaks at around 4 h after nebulization, whereas the maximum migration into interstitial and alveolar spaces occurs at around 12 to 24 h postnebulization (55). Engrafted 7-wk-old MISTRG and MISTRGGR^−/−^ mice were exposed to aerosolized LPS and killed at 24 h after nebulization. Bronchoalveolar lavage fluid (BALF), and lung tissues were harvested for flow cytometric assessment of infiltrating neutrophils and other immune cells (Fig. 5*A*). In order to differentiate immune cells residing in the lung vasculature from cells in the interstitium, we adopted a strategy of in vivo labeling of circulating human immune cells (56). Intravenous injection of anti-human CD45 allowed distinguishing between interstitial and intravascular neutrophils (*SI Appendix*, Fig. S5*A*).

At steady state, there were few human neutrophils in the lung of both MISTRGGR^−/−^ and MISTRG mice, although the numbers for MISTRGGR^−/−^ mice are significantly higher than MISTRG (Fig. 5*B*). However, upon LPS nebulization, there was a marked increase in human neutrophils in both intravenous and interstitial lung compartments in MISTRGGR^−/−^ mice with respect to control MISTRG mice in these same compartments (Fig. 5*B*). Although there was robust recruitment of mouse neutrophils in control MISTRG mice, human neutrophils failed to respond or expand (Fig. 5*B*). The recruitment of human neutrophils in MISTRGGR^−/−^ was even more dramatic in the BAL (Fig. 5*C*). The morphologies of cells isolated from BAL were further confirmed by MGG staining. In contrast to steady state, where neutrophils are absent, BAL isolates from LPS-treated MISTRGGR^−/−^ mice are enriched with mature hypersegmented human neutrophils (Fig. 5*D*). The accumulation of human neutrophils in the lung and BAL were accompanied by a significant reduction of CD101^+^ neutrophil (Neu) population in the bone marrow, suggesting a release of postmitotic neutrophils in response to LPS stimuli (Fig. 5*E*). The levels of circulating human and mouse neutrophils and other human immune lineages in the blood remained unaffected by LPS nebulization (*SI Appendix*, Figs. S2*B* and S5 *B*–*D*). Human neutrophils in MISTRGGR^−/−^ mice are recruited into the pulmonary vasculature (Fig. 5*B*), migrate first into the lung interstitium (Fig. 5*C* and *SI Appendix*, Fig. S5*A*), and then subsequently into the alveolar space (Fig. 5*D* and *SI Appendix*, Fig. S5*E*). Overall, these observations demonstrate that human neutrophils generated in MISTRGGR^−/−^ mice are able to develop and respond robustly to inflammatory stimuli by homing to the sites of inflammation.

### Reconstituted Human Neutrophils Respond to *P. aeruginosa* Infection in the Lung.

**Fig. 6.**
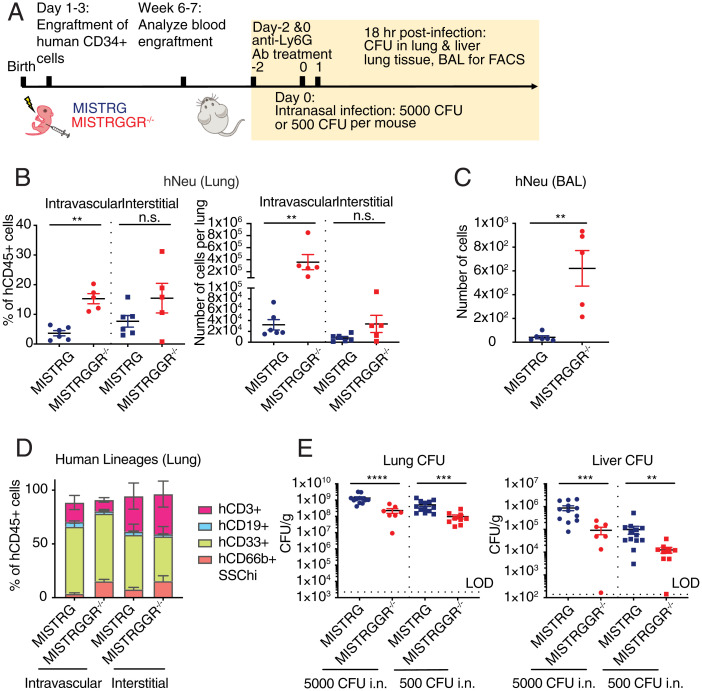
Human neutrophils in MISTRGGR mice provide protection against *P. aeruginosa* infection. (*A*) Experimental timeline of intranasal *P. aeruginosa* infection. Engrafted 7- to 8-wk-old MISTRG and MISTRGGR^−/−^ mice were pretreated with anti-mouse Ly6G antibody (1A8) (two doses of 100 µg, intravenously) for depletion of murine neutrophils, and then intranasally infected with 500 or 5000 CFU of *P. aeruginosa*. Lungs and livers were harvested for CFU quantification and flow cytometric characterization at 18 h after infection. (*B* and *C*) Frequencies and numbers of human neutrophils in the lung (*B*) and BAL (*C*) of infected mice (500 CFU, 18 h postinfection) (MISTRG, *n* = 6; MISTRGGR^−/−^, *n* = 5 mice). Data pooled from at least two independent experiments. (*D*) Frequencies of human lineages (MISTRG, *n* = 6; MISTRGGR^−/−^, *n* = 5 mice) in intravascular and interstitial lung postinfection (500 CFU, 18 h postinfection). (*E*) Bacterial load in lung (*Left*) and liver (*Right*) at 18 h after infection (5,000 CFU MISTRG, *n* = 12; MISTRGGR^−/−^, *n* = 7; 500 CFU MISTRG, *n* = 13; MISTRGGR^−/−^, *n* = 9 mice). Data pooled from at least three independent experiments. (*B*–*E*) Data are shown as mean ± SEM. *P* values determined by two-tailed Mann–Whitney test (*P < 0.05; **P < 0.01; ***P < 0.001; ****P < 0.0001). Each dot represents an individual mouse.

Finally, we tested the ability of human neutrophils to contain an infection, one of the major hallmarks of neutrophil function. *P. aeruginosa*, a gram-negative bacterium, is an opportunist pathogen that predominately infects neutropenic or immunocompromised individuals, such as cystic fibrosis patients, making it one of the leading causes of hospital-acquired pneumonia (25, 57). The clearance of *P. aeruginosa* during pulmonary infection is largely dependent on neutrophils and their intact machinery of intracellular killing following phagocytosis and extracellular killing following NET formation (25, 58, 59). Given the relevance of *Pseudomonas* infections in humans, we infected MISTRGGR^−/−^ and control MISTRG mice with *P. aeruginosa* intranasally (with a low and high dose of inoculation). In this experiment MISTRGGR^−/−^ and MISTRG mice were engrafted with fetal liver cells and were cohoused prior to infection to eliminate potential impacts of the microbiota. In order to assess the function of human neutrophils exclusive of mouse neutrophils, prior to infection, we depleted the remaining mouse neutrophil compartment by administrating anti-mouse Ly6G antibody intravenously (Fig. 6*A*). The degree of depletion in blood and lung were validated by flow cytometry (*SI Appendix*, Fig. S6*A*). Depletion of murine neutrophils by anti-Ly6G antibody also made MISTRG mice more susceptible to *P. aeruginosa* infection even at low doses of inoculation (*SI Appendix*, Fig. S6 *B* and *C*). Cell compositions in lung vasculature, interstitium, and BAL were assessed similarly by flow cytometry, as previously stated (*SI Appendix*, Fig. S5*A*).

In all of these compartments, human neutrophils in MISTRGGR^−/−^ greatly outnumbered human neutrophils in MISTRG mice (Fig. 6 *B* and *C*). MISTRGGR^−/−^ mice also had overall more human immune cells, but the composition of other immune cells except for granulocytes were comparable (Fig. 6*D*). Bacteria from lung and liver homogenates were plated and counted as an assessment of the severity of the infection and bacterial dissemination. MISTRGGR^−/−^ were able to better control *P. aeruginosa* infection, as suggested by lower bacterial loads in both lung and liver of MISTRGGR^−/−^ mice (Fig. 6*E*). Yet, the presence of human neutrophils did not completely clear the infection. To further elucidate that this was not due to functional impairment, we tested the phagocytic function of human neutrophils from MISTRGGR^−/−^ mice in an in vitro assay in the context of *P. aeruginosa* infection. Human neutrophils from humanized MISTRGGR^−/−^ mice internalized a higher proportion of *P. aeruginosa* compared with mouse neutrophils from MISTRGGR^−/−^ mice or human neutrophils (Fig. 3*B*). These findings suggest that failure to clear bacterial infection is a quantitative deficiency, as when numbers of matched human neutrophils from MISTRGGR^−/−^ mice outperform mouse neutrophils. Therefore, these data show that human neutrophils generated in MISTRGGR^−/−^ mice are functional and capable of responding to and reducing bacterial burden. Overall, MISTRGGR^−/−^ mice enable the development of functional, mature human neutrophils that are recruited during pulmonary infection to fulfill their essential role of fighting bacteria in vivo.

### Reconstituted Human Neutrophils Respond to Skin Inflammation.

It is well known that the recruitment of neutrophils into the lung is not dependent on the canonical adhesion pathways (60). Thus, we were interested in testing whether our model could be used to study other organs and tissues, such as the skin, that primarily utilize selection and integrin-mediated adhesion. To achieve this goal, we adopted a model of skin inflammation by tape-stripping, a surrogate for scratching, which induces mechanical insults and initiates neutrophil accumulation in mouse skin (*SI Appendix*, Fig. S7*A*) (61). We analyzed immune cell compositions of both control (untreated) and tape-treated ear at 24 h after tape-stripping. As compared to steady state, where very few human and mouse neutrophils were present, tape-stripping induced a substantial amount of influxes of both human and mouse neutrophils into the treated ear (*SI Appendix*, Fig. S7 *B* and *C*). The recruitment of human neutrophils is very prominent in some individual MISTRGGR^−/−^ mice, where the steady-state levels of human neutrophils in the bone marrow and skin are also higher (*SI Appendix*, Fig. S7 *B* and *C*). Our results have shown that the MISTRGGR model can support integrin-mediated infiltration of human neutrophils into the skin, proving this model to be useful in tissues outside of the lung.

## Discussion

Humanized mouse models have greatly expanded our understanding of how the human immune system works, and notably, have aided in translating findings in mice to humans. However, until now, there were no humanized mouse models that supported mature functional neutrophils, which are a cornerstone of the innate immune response. MISTRGGR^−/−^ mice described here overcome this major limitation in current generation humanized models by greatly improving human neutrophil numbers in blood and peripheral tissues. Importantly, this model allows functional integration of human neutrophil function in vivo and greatly expands the utility of humanized mice for studying complex human diseases.

In our system, the humanization of G-CSF alone did not improve human neutrophils. Results achieved by deletion of G-CSFR argue that this is due to cross-reactivity of mouse G-CSFR with human G-CSF, which display relatively high homology to mouse G-CSF (43). Given that murine granulocytes and precursors are still in the vast majority during the early weeks of engraftment, the presence of murine cells will likely have a competitive advantage over the human G-CSF and occupy niche space for granulopoiesis. Therefore, G-CSFR deletion was necessary to limit murine granulopoiesis and to allow human granulocyte development. This model still exhibits deficiencies. Given their additional deficiencies in murine neutrophils, MISTRGGR^−/−^ mice are highly immunocompromised and fragile; their life span is determined by proper care and housing conditions. They can live for an experimentally useful 14 wk postengraftment depending on the chimerism level of human hematopoietic and immune cells. Although MISTRGGR^−/−^ mice have significantly fewer mouse neutrophils, G-CSFR deficiency did not eliminate mouse neutrophils, opening up possibilities for further improvement of the existing system. Additionally, at steady state, human neutrophil numbers seen in the blood of MISTRGGR^−/−^ mice are still not comparable to the high proportions and numbers of neutrophils seen in human blood. We have confirmed a pool of postmitotic mature human neutrophils in the bone marrow of engrafted MISTRGGR^−/−^ mice (Fig. 4*C*), and yet these neutrophils display lower CD10 expression, a marker for mature human bone marrow neutrophils (53), as compared to mature neutrophils isolated from adult human bone marrow (*SI Appendix*, Fig. S4 *C* and *D*) (53). Nevertheless, our model is a major step forward to understanding why and will allow more mechanistic studies to be performed to understand how this could be achieved and what additional unknown factors are required for human neutrophil development.

Although human neutrophils effectively home to the lungs and are functional in reducing bacterial burden during *P. aeruginosa* infection, the presence of human neutrophils does not completely clear the infection. MISTRG mice, without depletion of murine neutrophils, have a lower bacterial load compared with engrafted MISTRGGR^−/−^ mice. This is not likely to be due to an inherent deficiency in the phagocytic potential of human macrophages but rather a result of a quantitative deficiency, as there are still fewer human neutrophils in engrafted MISTRGGR^−/−^ than there are mouse neutrophils in MISTRG mice (Fig. 6*B* and *SI Appendix*, Fig. S6*C*). Other antimicrobial, qualitative functions of infiltrating human neutrophils, beyond phagocytosis, bacterial killing, ROS production, and NET formation, and their cross-talk with other immune cells and molecules (e.g., antibodies) during infection remain elusive, some of which could potentially require further support in the humanized host.

A human immune system that supports mature neutrophils can be combined with various disease models to study the human-specific contribution of neutrophils during disease. Epidemics of drug-resistant and opportunist pathogens have emphasized the need to further investigate the interactions between human neutrophils and pathogens. *C. auris*, a recently emerged fungal pathogen posing global public health threats, has been shown to be capable of resisting human neutrophil killing by avoiding effective engagement, phagocytosis, as well as inhibiting the formation of NETs (26). Moreover, in contrast to the inability of human neutrophils to recognize and kill the pathogen, murine neutrophils appear to be able to control *C. auris* infection (62–64), suggesting a species-specific mechanism by which *C. auris* are able to resist human neutrophil killing. Given that our knowledge of neutrophil responses to infection has been largely derived from animal models and in vitro studies, understanding the in vivo action of human neutrophils in various bacterial and fungal infections remains critical. Here, we have demonstrated the potential to use MISTRGGR^−/−^ mice to study the interactions between human neutrophils and respiratory pathogens in the native lung environment.

In addition to the antimicrobial properties of neutrophils, there has been a growing appreciation of neutrophils in tumorigenesis. However, the present understanding of their roles remains under debate, as tumor-associated neutrophils, upon activation display both pro and antitumor properties (23, 32, 33, 65–70). It remains unclear whether differential programs for neutrophils (similar to M1 and M2 for macrophages) in the tumor environment truly exist, given that the heterogeneity observed could be due to differential states of activation and maturation (71, 72). To date, the majority of experimental approaches for studying neutrophils in tumor tissues have been limited to mouse models, as well as correlative studies of human neutrophils in various patient tumors. Thus, our model provides a unique possibility to cotransplant the human immune system and human tumors, which will allow the study of the role of neutrophils in tumor evolution and help to identify potential therapeutic targets.

Our humanized model that targets the cytokine–receptor pair of G-CSF significantly improves functional human neutrophil presence in the periphery but also highlights the importance of multiple strategies that are still needed to fully recapitulate functional human immune response. MISTRGGR^−/−^ mice are an important and necessary step forward in pushing the next generation of humanized mouse systems and provide a unique tool to study human neutrophils in vivo in presence of a more complete human immune system. Together, this in turn should enable the application of humanized mice to unexplored and wider areas of translational research.

## Materials and Methods

### Mice.

The generation of G-CSF knockin mice was performed on a MISTRG background with CRISPR guide designed to target region (chr11:98,701,464-98,701,486) in intron 1 of mouse *G-CSF*. Guide sequence: GGTCTGAGGCACTTGTTATCCGG. A construct containing human *G-CSF* ORF (1,485 bp) flanked by two homologous arms were provided. The resulting founding mouse contains the entire human ORF with murine 5′ and 3′ UTRs. The knockout of *G-CSFR* were conducted on MISTRGG mice with CRISPR guides designed to target both the ATG start codon and the beginning of 3′ UTR. Guide #1 sequences: GCTGGCAAAATGGTAGGGCTGGG. Guide #2 sequences: GAAATTCTGGCTGGACGTGGTGG. The resulting founding mouse contains a deletion of 16,798 bp (mChr4:126,027,350-126,044,148), starting immediately after start codon and 310 bp into 3′ UTR of G-CSFR. All mice were maintained under specific pathogen-free conditions with continuous treatment with enrofloxacin in the drinking water (Baytril, 0.27 mg/mL). MISTRGGR mice rely on prophylactic antibiotics due their immunocompromised state particularly prior to development of the human immune system. Reduced breeding capabilities in MISTRGGR females may pose difficulties in colony expansion, particularly when proper care and clean housing conditions are not maintained. For human cell engraftment, Sirpa m/m females and h/h males (e.g., MITRG females and MISTRG males) or Sirpa m/m males and h/h females (e.g., MITRG males and MISTRG females) were set up as breeding pairs to generate Sirpa h/m progenies, which were later engrafted with human CD34^+^ cells. All animal experimentations were performed in compliance with Yale Institutional Animal Care and Use Committee protocols. MISTRGGR mice will be made available either from Yale and deposited in a repository and to academic, nonprofit, and governmental institutions under a Yale-Regeneron-Howard Hughes Medical Institute material transfer agreement (already approved and agreed to by all parties). Instructions on obtaining the material transfer agreement for this mouse strain will be available along with strain information upon request.

### Human CD34^+^ Hematopoietic Stem and Progenitor Cell Isolation and Injection.

Human CD34^+^ hematopoietic stem and progenitor cells were isolated and injected into recipient mice as previously described (35). De-identified human fetal tissues were obtained from Advance Bioscience Resources. Briefly, human fetal liver samples were cut in small fragments, treated for 45 min at 37 °C with collagenase D (Roche; 200 μg/mL), and prepared into a cell suspension. Human CD34^+^ cells were purified from the single-cell suspension by performing density gradient centrifugation (Lymphocyte Separation Medium, MP Biomedicals), followed by positive immunomagnetic selection with EasySep Human CD34 Positive Selection Kit (Stemcell). Isolated cells were then counted and checked for CD34^+^ purity. Cells were frozen in FBS containing 10% DMSO and kept in liquid nitrogen for later use.

For intrahepatic engraftment, frozen CD34^+^ cells were thawed and recounted in prewarmed IMDM medium supplemented with 2% FBS and resuspended in PBS in appropriate volume. Newborn 1- to 3-d-old pups were sublethally irradiated at a dose of 150 Rads (X-ray irradiation with X-RAD 320 irradiator), and unless otherwise specified, 20,000 fetal liver CD34^+^ cells in 20 μL of PBS were injected into the liver with a 22-gauge needle (Hamilton). In one specific experiment (Fig. 1 *D* and *E*), 20,000 cord blood CD34^+^ cells were used for engraftment. All animal studies were performed in accordance with the guidelines of the Office of Animal Research Support at Yale University. All use of human materials was approved by the Yale University Human Investigation Committee.

### Gene-Expression Analysis.

Total RNA was extracted from tissues using TRIzol reagent (Thermo Fisher Scientific) and the DirectZol RNA Miniprep Kit (Zymo Research). cDNA synthesis was performed using SuperScript III Reverse Transcriptase (Thermo Fisher Scientific). qRT-PCR was performed using a CFX96 Real-Time PCR System (Bio-Rad) and iTaq Universal Probes Supermix (Bio-Rad). Sequence-specific oligonucleotide primers were purchased from Sigma-Aldrich. The following primers were used: human CSF3 (Hs00738432_g1), mouse Csf3 (Mm00438334_m1), and mouse Csf3r (Mm00432735_m1). Expression values were calculated using the comparative threshold cycle method and normalized to mouse Hprt (Mm00446968_m1).

### G-CSF Protein Quantification.

Mice were injected intraperitoneally with 50 μg of LPS and killed 2 or 3 h postinfection as detailed in the figure legends. Human (Sigma #RAB0103-1KT) and mouse (Sigma #RAB0104-1KT) G-CSF levels were quantified by ELISA in serum, bone marrow aspirates, lung homogenates (0.1 g), sorted endothelial cells, and sorted epithelial cells. Single-cell suspensions from lung and liver were stained with CD31-PE (BioLegend) and EPCAM-APC (BioLegend). Endothelial cells and epithelial cells were sorted using Sony SH800 Cell Sorter (100,000 cells). All tissue samples and cells were lysed in 1× RIPA buffer to allow release of G-CSF from cells. These lysates were spun at 10,000 × *g* for 10 min. Supernatant was used in G-CSF protein quantification at least in duplicates. ELISA kits used could detect G-CSF levels as low as 1 pg/mL.

### Characterization of Human Immune Cells by Flow Cytometry.

All mice were analyzed at ∼6 to 8 wk postengraftment. Blood was collected retro-orbitally in heparinized collection tubes. Heparinized samples were stained by an antibody mixture at room temperature for 20 min and then treated with red blood cell (RBC) lysis/fixation solution (BioLegend #422401). Fixed cells were then washed and resuspended with PBS supplemented with 2% FBS and 1 mM EDTA (FACS buffer) for analysis. Bone marrows (femurs and tibias) were dissected and flushed with FACS buffer through a 30-gauge needle (BD Biosciences) to form single-cell suspensions, which were subsequently treated with ammonium-chloride-potassium (ACK) lysis buffer to eliminate RBCs. Lung tissues were first chopped into small pieces, digested in RPMI1640 medium supplemented with collagenase D (1 mg/mL) for 35 min at 37 °C, and then filtered through 70-μm cell strainers (Fisher Scientific #07-201-431) to form single-cell suspensions.

Single-cell suspensions of bone marrow, lungs, and BALs were stained with antibody mixture on ice for 20 min and then washed with FACS buffer and fixed with Foxp3/Transcription Factor Fixation Buffer (eBioscience #00-5523-00) overnight. Fixed cells were then washed and resuspended with FACS buffer for analysis. Antibodies against the following antigens were used at final concentration of 0.5 μg/mL. Mouse antigens: FITC anti-mouse CD45 antibody (BioLegend #103108, clone: 30-F11), Alexa Fluor 700 anti-mouse CD45 antibody (BioLegend #103128, clone: 30-F11), APC/Cy7 anti-mouse Ly-6G antibody (BioLegend #127624, clone: 1A8), Alexa Fluor 488 anti-mouse Ly-6C antibody (BioLegend #128022, clone: HK1.4). Human antigens: APC/Cy7 anti-human CD3 antibody (BioLegend #300318, clone: HIT3a), PerCP/Cyanine 5.5 anti-human CD3 antibody (BioLegend #300328, clone: HIT3a), APC/Cyanine 7 anti-human CD10 antibody (BioLegend #312212, Clone: HI10a), PerCP/Cyanine 5.5 anti-human CD10 antibody (BioLegend #312216, clone: HI10a), APC/Cyanine 7 anti-human CD14 antibody (BioLegend #325620, clone: HCD14), Pacific blue anti-human CD14 antibody (BioLegend #325616, clone: HCD14), Pacific blue anti-human CD15 (SSEA-1) antibody (BioLegend #323024, clone: W6D3), FITC anti-human CD16 antibody (BioLegend #302006, clone: 3G8), PE/Cy7 anti-human CD19 antibody (BioLegend #302216, clone: HIB19), APC anti-human CD33 antibody (BioLegend #303408, clone: WM53), APC/Cyanine 7 anti-human CD34 antibody (BioLegend #343614, clone: 561), PerCP/Cyanine 5.5 anti-human CD34 antibody (BioLegend #343612, clone: 561), APC/Cyanine 7 anti-human CD45 antibody (BioLegend #304014, clone: HI30), FITC anti-human CD45 antibody (BioLegend #304006, Clone: HI30), Pacific blue anti-human CD45 antibody (BioLegend #304022, clone: HI30), Brilliant violet 605 anti-human CD45 antibody (BioLegend #304042, Clone: HI30), APC anti-human CD49d antibody (BioLegend #304308, clone: 9F10), APC anti-human CD66b antibody (BioLegend #305118, clone: G10F5), PE anti-human CD66b antibody (BioLegend #305106, clone: G10F5), PerCP/Cyanine 5.5 anti-human CD66b antibody (BioLegend #305108, clone: G10F5), PE/Cy7 anti-human CD101 (BB27) antibody (BioLegend #331014, Clone: BB27), Pacific blue anti-human CD335 (NKp46) antibody (BioLegend #331912, clone: 9E2), PE anti-human CD335 (NKp46) antibody (BioLegend #331908, clone: 9E2). Data were acquired with FACSDiva 7 on an LSRII flow cytometer (BD Biosciences) and analyzed with FlowJo v10 software.

### Chemotaxis.

Human neutrophils were isolated by positive selection using EasySep protocols according to manufacturer’s instructions. The EZ-TAXIScan (ECI Frontier, MIC-1000) was used to investigate chemotaxis of human neutrophils. After removal of bubbles and heating of the chamber to 37 °C, 10,000 neutrophils in 1.5 μL of HBSS supplemented with 0.1% endotoxin-free BSA were loaded into the lower reservoir. Next, 1 μL of 1 μM IL-8 was loaded into the upper reservoir. Time-lapse images were taken for 20 min at 30-s intervals. At least 20 cell tracks from each sample were analyzed using Gradientech Tracking Tool PRO v2.1 (free software). Chemotaxis speed and directionality toward fMLP were determined by the cell tracks. Upward directionality was calculated based on the center of mass and accumulated distance.

### In Vitro Phagocytosis Assays.

Protocols as previously described (51) were adapted and optimized. Mice were bled retro-orbitally as previously described. Blood was incubated with pHrodo Green *E. coli* BioParticles Conjugate (Life Technologies; 1 mg/mL) in a 96-well plate at 37 °C for 0, 20, and 90 min, respectively (10 μg *E.coli* particles per 50 μL blood). After incubation, cells were fixed at an added final concentration of 1.6% PFA. Cells were stained for lineage characterization as previously described. All samples were lysed and fixed with RBC lysis and fixation buffer (BioLegend). All buffers were tested for pH 7.4 prior to use.

For *P. aeruginosa* killing assays, human neutrophils were isolated by positive selection as described for chemotaxis assays. Mouse neutrophils were isolated by density-gradient centrifuging with Histopaque 1119 and 1077, as described in previously (73). Midlog cultures of *P. aeruginosa* were washed and opsonized with 10% fresh human serum for 20 min at 37 °C. Equal numbers of human and mouse neutrophils (100,000 cells per well) were cultured in HBSS with live *P. aeruginosa* at a multiplicity of infection of 1:10 in 37 °C for 30 min. After removal of culture supernatant, neutrophils were washed and incubated with 400 μg/mL gentamicin for 30 min at 37 °C to kill external bacteria, and subsequently lysed with 0.1% Triton X-100. Culture supernatant and cell lysate were then diluted and plated on LB plates and cultured at 37 °C overnight. Bacterial counts and frequencies of internalized bacteria were counted from culture supernatant and cell lysate separately.

### ROS Production Assay.

Bone marrow human neutrophils and mouse neutrophils were isolated as described for previous assays. Blood was drawn as described above. Isolated bone marrow neutrophils or whole blood were stimulated with PMA (10 ng/mL) for 1 or 2 h at 37 °C, or by human TNF-α (20 ng/mL) for 30 min, and stimulation with human fMLP (10 μM) for 30 min at 37 °C. Cells were washed and stained with CellROX Green agents at 500 nM (Invitrogen). Cells were then subsequently stained with Fixable Cell Viability Dye (eFlour506, eBioscience) and antibody panel. ROS production was analyzed by flow cytometry and mean fluorescence intensity (MFI) was calculated.

### NETosis Assay.

Isolated human and mouse neutrophils from engrafted bone marrow were cultured unstimulated or with 20 nM PMA for 4 h at 37 °C. MPO and histone H3 were detected by flow cytometry using previously published methods (74). NETs-forming neutrophils are both MPO^+^ and H3Cit^+^.

### LPS Nebulization, In Vivo Labeling, and Tissue Collection.

Mice were exposed to aerosolized LPS (from *P. aeruginosa* 10, Sigma L9143) using a nebulizer (Pulmo-Aide compressor) as previously described (75). LPS (12.5 mg) dissolved in 5.0 mL of PBS were administered using a nebulizer connected to a container with vent holes. Five milliliters of solution were administered over 15 min.

At 24 h after nebulization, mice were anesthetized. To distinguish between circulating and lung resident or infiltrating human immune cells, fluorochrome labeled (FITC or APC/Cy7) anti-human CD45 antibody (2.5 μg, clone HI30) was intravenously injected. Mice were kept under anesthesia for at least 5 min after injection. Blood samples were collected retro-orbitally and BAL was performed using standard methods with a 22-gauge catheter (BD Biosciences) (76). Lungs were then aspirated with sterile PBS. Mice were then killed for bone marrow and lung collection. All tissues were processed, stained, and analyzed as previously described.

### *P. aeruginosa* Infection.

To deplete mouse neutrophils in recipient mice, two doses of 100 μg anti-Ly6G antibody (clone: 1A8, BioXCell) in 100 μL PBS were intravenously injected at ∼48 h and 3 h prior to the intranasal admiration of *P. aeruginosa*. Rat IgG2a antibody (BioXCell) was used as isotype control.

*P. aeruginosa* (PA14 strain provided by B. Kaczmierczek, Yale University, New Haven, CT) was maintained as a glycerol stock at −80 °C. Prior to inoculation (∼24 h), bacteria were grown at 37 °C in a shaking incubator for ∼16 h in LB medium. Bacteria were subcultured in a 1:20 ratio in a volume of 20 mL until OD_600_ was between 1.5 and 2.0, which is ∼1.5 × 10^9^ and 2 × 10^9^ CFU/mL bacteria were washed with PBS and centrifuged twice (8,000 rpm, 2 min), and diluted to an appropriate volume. Mice were anesthetized with methoxyflurane, and a dose of 500 or 5,000 CFU in 20 μL PBS was instilled intranasally using a micropipette.

Mice were killed at 18 or 24 h after infection, unless otherwise stated. Livers and lungs were collected sterilely and homogenized with gentleMACS Dissociator (Miltenyi). Tissue homogenates were then diluted and plated on LB plates and cultured at 37 °C overnight. Bacterial loads were calculated from CFU counts. Alternately, a subset of mice was used to characterize of the immune cells by flow cytometry. In vivo labeling of human immune cells was performed, and lungs were processed as previously described.

### Ear Tape-Stripping Model.

Under anesthesia, the left ears of the mice were left as control, while the right ears were repeatedly tape-stripped eight times on both sides. Both ears were collected for analysis of immune composition 24 h after tape-stripping. Ears were peeled into two pieces and cut into small pieces. Each ear was digested in 1.2 mL of RPMI supplemented with 0.4 mg/mL liberase TM and 60 ng/mL DNaseI at 37 °C for 45 min. After digestion, cells were filtered through a 70-µm strainer and stained as other tissues.

### Cell Morphology.

Cells sorted from bone marrow or isolated from BAL were cytospun onto slides at 200 rpm for 5 min (∼100,000 cells per slide) and stained with MGG (Sigma) following the manufacturer’s instructions. Blood smears were performed using standard methods and stained with MGG.

### Statistical Analysis.

Statistical analysis was performed with the GraphPad Prism 7 software, using two-tailed unpaired Student’s *t* test (Mann–Whitney test).

## Supplementary Material

Supplementary File

Supplementary File

Supplementary File

Supplementary File

Supplementary File

## Data Availability

All study data are included in the main text and supporting information.

## References

[1] J. Mestas, C. C. Hughes, Of mice and not men: Differences between mouse and human immunology. J. Immunol. 172, 2731–2738 (2004). 14978070 10.4049/jimmunol.172.5.2731

[2] D. L. Gibbons, J. Spencer, Mouse and human intestinal immunity: Same ballpark, different players; different rules, same score. Mucosal Immunol. 4, 148–157 (2011). 21228770 10.1038/mi.2010.85

[3] A. Rongvaux , Human hemato-lymphoid system mice: Current use and future potential for medicine. Annu. Rev. Immunol. 31, 635–674 (2013). 23330956 10.1146/annurev-immunol-032712-095921 PMC4120191

[4] J. Zschaler, D. Schlorke, J. Arnhold, Differences in innate immune response between man and mouse. Crit. Rev. Immunol. 34, 433–454 (2014). 25404048

[5] T. Shay ; ImmGen Consortium, Conservation and divergence in the transcriptional programs of the human and mouse immune systems. Proc. Natl. Acad. Sci. U.S.A. 110, 2946–2951 (2013). 23382184 10.1073/pnas.1222738110 PMC3581886

[6] L. G. Ng, R. Ostuni, A. Hidalgo, Heterogeneity of neutrophils. Nat. Rev. Immunol. 19, 255–265 (2019). 30816340 10.1038/s41577-019-0141-8

[7] L. S. C. Lok , Phenotypically distinct neutrophils patrol uninfected human and mouse lymph nodes. Proc. Natl. Acad. Sci. U.S.A. 116, 19083–19089 (2019). 31484769 10.1073/pnas.1905054116 PMC6754587

[8] M. Casanova-Acebes , Neutrophils instruct homeostatic and pathological states in naive tissues. J. Exp. Med. 215, 2778–2795 (2018). 30282719 10.1084/jem.20181468 PMC6219739

[9] E. Kolaczkowska, P. Kubes, Neutrophil recruitment and function in health and inflammation. Nat. Rev. Immunol. 13, 159–175 (2013). 23435331 10.1038/nri3399

[10] W. M. Nauseef, N. Borregaard, Neutrophils at work. Nat. Immunol. 15, 602–611 (2014). 24940954 10.1038/ni.2921

[11] P. Scapini , The neutrophil as a cellular source of chemokines. Immunol. Rev. 177, 195–203 (2000). 11138776 10.1034/j.1600-065x.2000.17706.x

[12] O. Zöllner , L-selectin from human, but not from mouse neutrophils binds directly to E-selectin. J. Cell Biol. 136, 707–716 (1997). 9024699 10.1083/jcb.136.3.707 PMC2134294

[13] E. Aleyd, M. H. Heineke, M. van Egmond, The era of the immunoglobulin A Fc receptor FcαRI; its function and potential as target in disease. Immunol. Rev. 268, 123–138 (2015). 26497517 10.1111/imr.12337

[14] E. Hajjar, T. Broemstrup, C. Kantari, V. Witko-Sarsat, N. Reuter, Structures of human proteinase 3 and neutrophil elastase—So similar yet so different. FEBS J. 277, 2238–2254 (2010). 20423453 10.1111/j.1742-4658.2010.07659.x

[15] M. A. Otten , Immature neutrophils mediate tumor cell killing via IgA but not IgG Fc receptors. J. Immunol. 174, 5472–5480 (2005). 15843545 10.4049/jimmunol.174.9.5472

[16] C. Tecchio, A. Micheletti, M. A. Cassatella, Neutrophil-derived cytokines: Facts beyond expression. Front. Immunol. 5, 508 (2014). 25374568 10.3389/fimmu.2014.00508 PMC4204637

[17] N. Tamassia , Cutting edge: An inactive chromatin configuration at the IL-10 locus in human neutrophils. J. Immunol. 190, 1921–1925 (2013). 23355741 10.4049/jimmunol.1203022

[18] A. M. Condliffe , Sequential activation of class IB and class IA PI3K is important for the primed respiratory burst of human but not murine neutrophils. Blood 106, 1432–1440 (2005). 15878979 10.1182/blood-2005-03-0944

[19] J. Bagaitkar, J. D. Matute, A. Austin, A. A. Arias, M. C. Dinauer, Activation of neutrophil respiratory burst by fungal particles requires phosphatidylinositol 3-phosphate binding to p40(phox) in humans but not in mice. Blood 120, 3385–3387 (2012). 23086626 10.1182/blood-2012-07-445619

[20] T. Ganz, Defensins: Antimicrobial peptides of innate immunity. Nat. Rev. Immunol. 3, 710–720 (2003). 12949495 10.1038/nri1180

[21] P. B. Eisenhauer, R. I. Lehrer, Mouse neutrophils lack defensins. Infect. Immun. 60, 3446–3447 (1992). 1639513 10.1128/iai.60.8.3446-3447.1992 PMC257335

[22] P. G. Rausch, T. G. Moore, Granule enzymes of polymorphonuclear neutrophils: A phylogenetic comparison. Blood 46, 913–919 (1975). 173439

[23] E. B. Eruslanov, S. Singhal, S. M. Albelda, Mouse versus human neutrophils in cancer: A major knowledge gap. Trends Cancer 3, 149–160 (2017). 28718445 10.1016/j.trecan.2016.12.006 PMC5518602

[24] A. R. Witter, B. M. Okunnu, R. E. Berg, The essential role of neutrophils during infection with the intracellular bacterial pathogen listeria monocytogenes. J. Immunol. 197, 1557–1565 (2016). 27543669 10.4049/jimmunol.1600599 PMC4995063

[25] B. Rada, Interactions between neutrophils and *Pseudomonas aeruginosa* in cystic fibrosis. Pathogens 6, E10 (2017). 28282951 10.3390/pathogens6010010 PMC5371898

[26] C. J. Johnson, J. M. Davis, A. Huttenlocher, J. F. Kernien, J. E. Nett, Emerging fungal pathogen *Candida auris* evades neutrophil attack. MBio 9, e01403-18 (2018). 30131360 10.1128/mBio.01403-18 PMC6106086

[27] O. Jones-Nelson , The neutrophilic response to pseudomonas damages the airway barrier, promoting infection by *Klebsiella pneumoniae*. Am. J. Respir. Cell Mol. Biol. 59, 745–756 (2018). 30109945 10.1165/rcmb.2018-0107OC

[28] G. J. Lieschke , Mice lacking granulocyte colony-stimulating factor have chronic neutropenia, granulocyte and macrophage progenitor cell deficiency, and impaired neutrophil mobilization. Blood 84, 1737–1746 (1994). 7521686

[29] A. Triot , Inherited biallelic CSF3R mutations in severe congenital neutropenia. Blood 123, 3811–3817 (2014). 24753537 10.1182/blood-2013-11-535419 PMC4055927

[30] G. Morstyn , Effect of granulocyte colony stimulating factor on neutropenia induced by cytotoxic chemotherapy. Lancet 1, 667–672 (1988). 2895212 10.1016/s0140-6736(88)91475-4

[31] A. R. Russell, E. G. Davies, S. E. Ball, E. Gordon-Smith, Granulocyte colony stimulating factor treatment for neonatal neutropenia. Arch. Dis. Child. Fetal Neonatal Ed. 72, F53–F54 (1995). 7538031 10.1136/fn.72.1.f53 PMC2528420

[32] Z. G. Fridlender , Polarization of tumor-associated neutrophil phenotype by TGF-beta: “N1” versus “N2” TAN. Cancer Cell 16, 183–194 (2009). 19732719 10.1016/j.ccr.2009.06.017 PMC2754404

[33] H. Nozawa, C. Chiu, D. Hanahan, Infiltrating neutrophils mediate the initial angiogenic switch in a mouse model of multistage carcinogenesis. Proc. Natl. Acad. Sci. U.S.A. 103, 12493–12498 (2006). 16891410 10.1073/pnas.0601807103 PMC1531646

[34] I. Puga , B cell-helper neutrophils stimulate the diversification and production of immunoglobulin in the marginal zone of the spleen. Nat. Immunol. 13, 170–180 (2011). 22197976 10.1038/ni.2194 PMC3262910

[35] A. Rongvaux , Development and function of human innate immune cells in a humanized mouse model. Nat. Biotechnol. 32, 364–372 (2014). 24633240 10.1038/nbt.2858 PMC4017589

[36] M. J. Christopher, D. C. Link, Regulation of neutrophil homeostasis. Curr. Opin. Hematol. 14, 3–8 (2007). 17133093 10.1097/00062752-200701000-00003

[37] A. D. Panopoulos, S. S. Watowich, Granulocyte colony-stimulating factor: Molecular mechanisms of action during steady state and ‘emergency’ hematopoiesis. Cytokine 42, 277–288 (2008). 18400509 10.1016/j.cyto.2008.03.002 PMC2852428

[38] C. L. Semerad, F. Liu, A. D. Gregory, K. Stumpf, D. C. Link, G-CSF is an essential regulator of neutrophil trafficking from the bone marrow to the blood. Immunity 17, 413–423 (2002). 12387736 10.1016/s1074-7613(02)00424-7

[39] N. Strydom, S. M. Rankin, Regulation of circulating neutrophil numbers under homeostasis and in disease. J. Innate Immun. 5, 304–314 (2013). 23571274 10.1159/000350282 PMC6741587

[40] S. Boettcher , Endothelial cells translate pathogen signals into G-CSF-driven emergency granulopoiesis. Blood 124, 1393–1403 (2014). 24990886 10.1182/blood-2014-04-570762 PMC4148762

[41] M. G. Manz, S. Boettcher, Emergency granulopoiesis. Nat. Rev. Immunol. 14, 302–314 (2014). 24751955 10.1038/nri3660

[42] N. A. Nicola, Granulocyte colony-stimulating factor and differentiation-induction in myeloid leukemic cells. Int. J. Cell Cloning 5, 1–15 (1987). 3031177 10.1002/stem.5530050102

[43] N. A. Nicola, C. G. Begley, D. Metcalf, Identification of the human analogue of a regulator that induces differentiation in murine leukaemic cells. Nature 314, 625–628 (1985). 2986009 10.1038/314625a0

[44] F. A. Ran , Genome engineering using the CRISPR-Cas9 system. Nat. Protoc. 8, 2281–2308 (2013). 24157548 10.1038/nprot.2013.143 PMC3969860

[45] B. Bajrami , G-CSF maintains controlled neutrophil mobilization during acute inflammation by negatively regulating CXCR2 signaling. J. Exp. Med. 213, 1999–2018 (2016). 27551153 10.1084/jem.20160393 PMC5030805

[46] K. M. Zsebo , Recombinant human granulocyte colony stimulating factor: Molecular and biological characterization. Immunobiology 172, 175–184 (1986). 3492428 10.1016/S0171-2985(86)80097-3

[47] F. Liu, H. Y. Wu, R. Wesselschmidt, T. Kornaga, D. C. Link, Impaired production and increased apoptosis of neutrophils in granulocyte colony-stimulating factor receptor-deficient mice. Immunity 5, 491–501 (1996). 8934575 10.1016/s1074-7613(00)80504-x

[48] J. Pillay, T. Tak, V. M. Kamp, L. Koenderman, Immune suppression by neutrophils and granulocytic myeloid-derived suppressor cells: Similarities and differences. Cell. Mol. Life Sci. 70, 3813–3827 (2013). 23423530 10.1007/s00018-013-1286-4 PMC3781313

[49] G. L. Burn, A. Foti, G. Marsman, D. F. Patel, A. Zychlinsky, The neutrophil. Immunity 54, 1377–1391 (2021). 34260886 10.1016/j.immuni.2021.06.006

[50] M. Casanova-Acebes , Rhythmic modulation of the hematopoietic niche through neutrophil clearance. Cell 153, 1025–1035 (2013). 23706740 10.1016/j.cell.2013.04.040 PMC4128329

[51] N. Fine, O. Barzilay, M. Glogauer, Analysis of human and mouse neutrophil phagocytosis by flow cytometry. Methods Mol. Biol. 1519, 17–24 (2017). 27815870 10.1007/978-1-4939-6581-6_2

[52] D. Hartl , Infiltrated neutrophils acquire novel chemokine receptor expression and chemokine responsiveness in chronic inflammatory lung diseases. J. Immunol. 181, 8053–8067 (2008). 19017998 10.4049/jimmunol.181.11.8053

[53] M. Evrard , Developmental analysis of bone marrow neutrophils reveals populations specialized in expansion, trafficking, and effector functions. Immunity 48, 364–379.e8 (2018). 29466759 10.1016/j.immuni.2018.02.002

[54] C. Liongue, C. Wright, A. P. Russell, A. C. Ward, Granulocyte colony-stimulating factor receptor: Stimulating granulopoiesis and much more. Int. J. Biochem. Cell Biol. 41, 2372–2375 (2009). 19699815 10.1016/j.biocel.2009.08.011

[55] J. Reutershan, A. Basit, E. V. Galkina, K. Ley, Sequential recruitment of neutrophils into lung and bronchoalveolar lavage fluid in LPS-induced acute lung injury. Am. J. Physiol. Lung Cell. Mol. Physiol. 289, L807–L815 (2005). 15951336 10.1152/ajplung.00477.2004

[56] A. G. Solis , Mechanosensation of cyclical force by PIEZO1 is essential for innate immunity. Nature 573, 69–74 (2019). 31435009 10.1038/s41586-019-1485-8 PMC6939392

[57] P. K. Mandal , Micro-organisms associated with febrile neutropenia in patients with haematological malignancies in a tertiary care hospital in eastern India. Indian J. Hematol. Blood Transfus. 31, 46–50 (2015). 25548444 10.1007/s12288-014-0393-1 PMC4275510

[58] E. G. Lavoie, T. Wangdi, B. I. Kazmierczak, Innate immune responses to *Pseudomonas aeruginosa* infection. Microbes Infect. 13, 1133–1145 (2011). 21839853 10.1016/j.micinf.2011.07.011 PMC3221798

[59] R. L. Young , Neutrophil extracellular trap (NET)-mediated killing of *Pseudomonas aeruginosa*: Evidence of acquired resistance within the CF airway, independent of CFTR. PLoS One 6, e23637 (2011). 21909403 10.1371/journal.pone.0023637 PMC3164657

[60] J. P. Mizgerd , Neutrophil emigration in the skin, lungs, and peritoneum: Different requirements for CD11/CD18 revealed by CD18-deficient mice. J. Exp. Med. 186, 1357–1364 (1997). 9334375 10.1084/jem.186.8.1357 PMC2199087

[61] M. K. Oyoshi , Leukotriene B4-driven neutrophil recruitment to the skin is essential for allergic skin inflammation. Immunity 37, 747–758 (2012). 23063331 10.1016/j.immuni.2012.06.018 PMC3478399

[62] B. Ferwerda , Human dectin-1 deficiency and mucocutaneous fungal infections. N. Engl. J. Med. 361, 1760–1767 (2009). 19864674 10.1056/NEJMoa0901053 PMC2773015

[63] X. Li , The β-glucan receptor Dectin-1 activates the integrin Mac-1 in neutrophils via Vav protein signaling to promote *Candida albicans* clearance. Cell Host Microbe 10, 603–615 (2011). 22177564 10.1016/j.chom.2011.10.009 PMC3244687

[64] R. Ben-Ami , Multidrug-resistant *Candida haemulonii* and *C. auris*, Tel Aviv, Israel. Emerg. Infect. Dis. 23, 195–203(2017). 10.3201/eid2302.161486 PMC5324804 28098529

[65] M. Albanesi , Neutrophils mediate antibody-induced antitumor effects in mice. Blood 122, 3160–3164 (2013). 23980063 10.1182/blood-2013-04-497446 PMC3814733

[66] A. M. Houghton , Neutrophil elastase-mediated degradation of IRS-1 accelerates lung tumor growth. Nat. Med. 16, 219–223 (2010). 20081861 10.1038/nm.2084 PMC2821801

[67] Z. Granot , Tumor entrained neutrophils inhibit seeding in the premetastatic lung. Cancer Cell 20, 300–314 (2011). 21907922 10.1016/j.ccr.2011.08.012 PMC3172582

[68] MET promotes antitumor neutrophil recruitment and cytotoxicity. Cancer Discov. 5, 689 (2015). 10.1158/2159-8290.CD-RW2015-097 26022610

[69] A. Blaisdell , Neutrophils oppose uterine epithelial carcinogenesis via debridement of hypoxic tumor cells. Cancer Cell 28, 785–799 (2015). 26678340 10.1016/j.ccell.2015.11.005 PMC4698345

[70] Y. Liu , CD11b+Ly6G+ cells inhibit tumor growth by suppressing IL-17 production at early stages of tumorigenesis. OncoImmunology 5, e1061175 (2015). 26942073 10.1080/2162402X.2015.1061175 PMC4760327

[71] J. M. Adrover, J. A. Nicolás-Ávila, A. Hidalgo, Aging: A temporal dimension for neutrophils. Trends Immunol. 37, 334–345 (2016). 27083489 10.1016/j.it.2016.03.005

[72] J. A. Nicolás-Ávila, J. M. Adrover, A. Hidalgo, Neutrophils in homeostasis, immunity, and cancer. Immunity 46, 15–28 (2017). 28099862 10.1016/j.immuni.2016.12.012

[73] M. Swamydas, Y. Luo, M. E. Dorf, M. S. Lionakis, Isolation of mouse neutrophils. Curr. Protoc. Immunol. 110, 3.20.1–3.20.15 (2015). 26237011 10.1002/0471142735.im0320s110 PMC4574512

[74] M. Gavillet , Flow cytometric assay for direct quantification of neutrophil extracellular traps in blood samples. Am. J. Hematol. 90, 1155–1158 (2015). 26347989 10.1002/ajh.24185 PMC4715743

[75] A. Taylor , SRF is required for neutrophil migration in response to inflammation. Blood 123, 3027–3036 (2014). 24574460 10.1182/blood-2013-06-507582 PMC4014845

[76] F. Sun, G. Xiao, Z. Qu, Murine bronchoalveolar lavage. Bio Protoc. 7, e2287 (2017). 10.21769/BioProtoc.2287 PMC5659634 29082285

