# Supplementary material for: Human neutrophil development and functionality are enabled in a humanized mouse model
Source: Proc Natl Acad Sci U S A. 2022 Oct 21;119(43):e2121077119. doi: 10.1073/pnas.2121077119 (PMC9618085; doi:10.1073/pnas.2121077119)
Supplement: Supplementary File [file pnas.2121077119.sapp.pdf]

PNAS

Supporting Information for

Human neutrophil development and functionality are enabled in a humanized mouse model.

Yunjiang Zheng[1, 13], Esen Sefik[1, 13], John Astle[1,2], Kutay Karatepe[3], Hasan H. Öz[4], Angel G. Solis[1, 5], Ruaidhrí Jackson[1,6], Hongbo R. Luo[7,8,9], Emanuela M. Bruscia[4], Stephanie Halene[10], Liang Shan[1, 11], and Richard A Flavell[1, 12*]

1 Department of Immunobiology, Yale University, New Haven, Connecticut, USA.
2 Department of Pathology, Medical College of Wisconsin, Milwaukee, WI, USA.
3 Department of Cell Biology, Yale Stem Cell Center, New Haven, Connecticut, USA
4 Section of Pediatric Pulmonology, Allergy, Immunology & Sleep Medicine, Department of Pediatrics, Yale University School of Medicine, New Haven, CT, USA.
5 Department of Microbiology, University of Pennsylvania, Philadelphia, Pennsylvania, USA.
6 Department of Immunology, Harvard Medical School, Boston, MA, USA.
7 Department of Laboratory Medicine, The Stem Cell Program, Boston Children's Hospital, Boston, MA, USA
8 Department of Pathology, Harvard Medical School, Boston, MA, USA
9 Dana-Farber/Harvard Cancer Center, Boston, MA, USA
10 Section of Hematology, Department of Internal Medicine and Yale Comprehensive Cancer Center, Yale University School of Medicine, New Haven, CT, USA.
11 Division of Infectious Diseases, Department of Medicine, Washington University School of Medicine, St. Louis, MO, USA.
12 Howard Hughes Medical Institute, Yale University, New Haven, Connecticut, USA.
13 These authors contributed equally to this work.
*Richard A Flavell

Richard A Flavell
Email: richard.flavell@yale.edu

This PDF file includes:

Figures S1 to S7
Legends for Movies S1 to S4

Other supporting materials for this manuscript include the following:

Movies S1 to S4

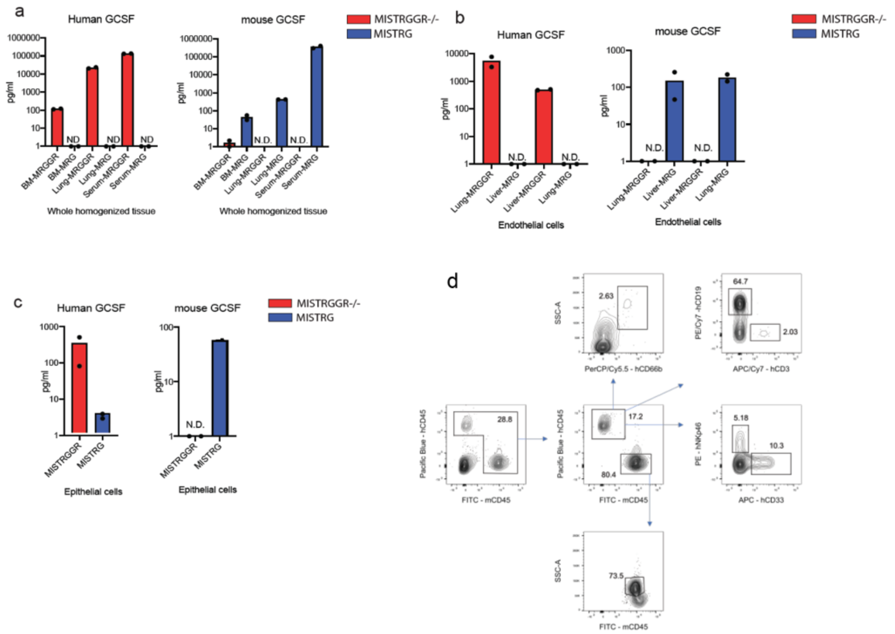

Fig. S1. Characterization of MISTRGGR$^{-/-}$ mice.
a. Human and mouse GCSF protein levels measured in the bone marrow (single femur), homogenized lung tissue (0.1g) and serum (100ul) of MISTRG and MISTRGGR$^{-/-}$ mice with LPS (50ug i.p) at 2 hours after LPS administration (50ug, i.p.).
b. Human and mouse GCSF protein levels measured in sorted lung and liver endothelial cells (CD31+) from MISTRG and MISTRGGR$^{-/-}$ mice with LPS (50ug I.P) at 2 hours after LPS administration (50ug, i.p.). For each data point, 100,000 cells were sorted and analyzed in duplicates.
c. Human and mouse GCSF protein levels measured in sorted lung epithelial cells (EPCAM+) from MISTRG and MISTRGGR$^{-/-}$ mice with LPS (50ug I.P) at 2 hours after LPS administration (50ug, I.p.). For each data point, 100,000 cells were sorted and analyzed in duplicates.
d. Characterization of human immune lineages in engrafted MISTRG mice. Representative flow plots for the gating strategy for human immune cell lineages in the blood are shown. Live single cells were first gated for a combined human(h) and mouse(m) CD45+, gate and then hCD45+ and mCD45+ respectively. hCD45+ cells were subsequently gated for hCD3+ (T cells), hCD19+ (B cells), hCD33+ (myeloid cells/monocytes), hCD66b+ SSChi (neutrophils), and hNKp46+ (NK cells). mCD45+ population were gated for SSChi (neutrophils). In later experiments, mouse neutrophils were also identified by mLy6G+, or Ly6Cmid SSChi.

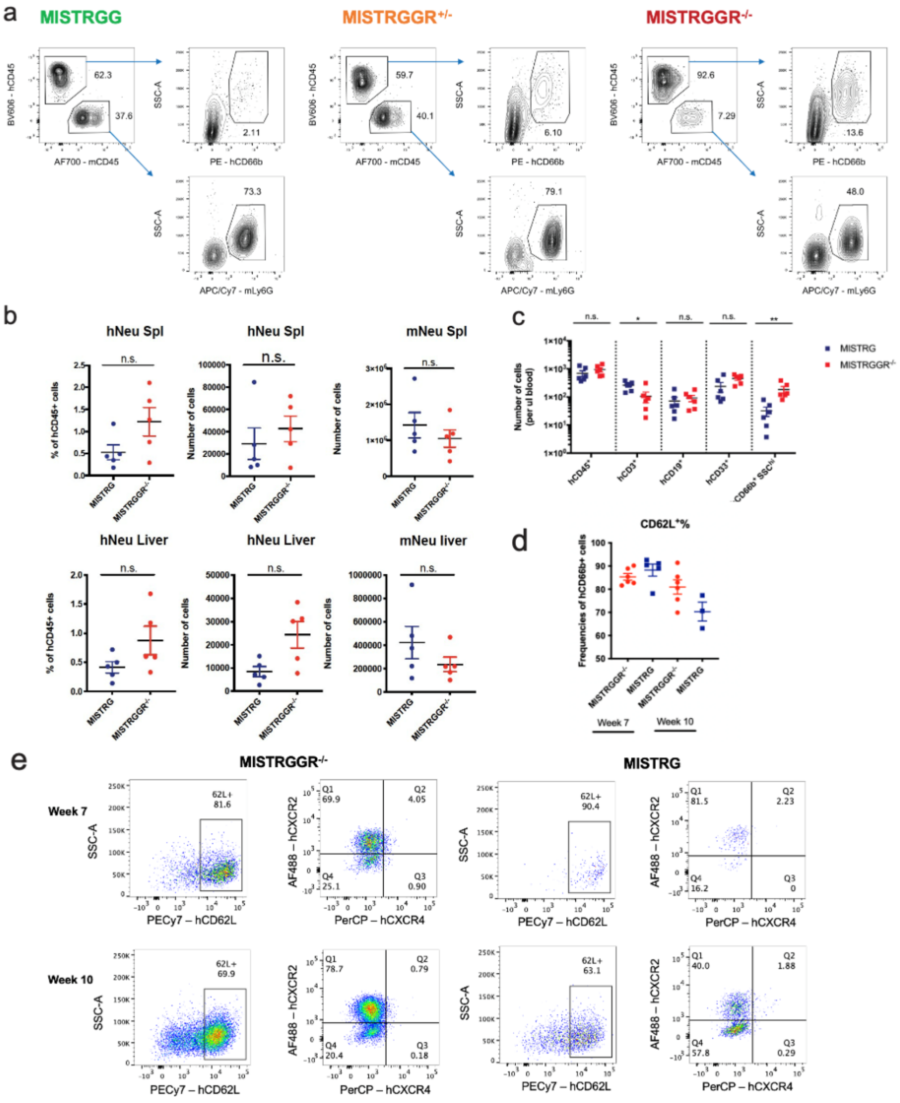

Fig. S2. Characterization of human immune cells in engrafted MISTRG and MISTRGGR$^{-/-}$ mice.

a. Representative flow cytometry gating of human neutrophils in the blood of engrafted MISTRG, MISTRGG, MISTRGGR+/- and MISTRGGR$^{-/-}$ mice at 7 weeks post-engraftment.

b. Frequencies and numbers of human (CD66b+) and mouse granulocytes (Ly6G+) in spleen (top) and liver (bottom) of MISTRG (n=5) and MISTRGGR+/- (n=5) mice.

c. Quantifications of human immune lineages in blood at week 7 post-engraftment (n=6).

d. Frequencies of ‘fresh’ neutrophils (CD62L+ CXCR2hi hCXCR4lo) among reconstituted human neutrophils in the blood of MISTRGGR$^{-/-}$ (n=6) and MISTRG (n=5) at week 7 and week 10 post-transplantation.

e. Representative flow cytometry plots of CD62L, CXCR2 and CXCR4 expression on reconstituted human neutrophils in the blood (gated on CD66b+ CD33lo neutrophils).

a-e, All mice were irradiated with 150 Rads and intra-hepatically injected with 10,000-30,000 human fetal liver CD34+ cells at 1-3 days after birth. Data were representative of at least 2 independent experiments. Data are shown as mean ± s.e.m.. P values determined by two-tailed Mann–Whitney test (*P < 0.05; **P < 0.01; ***P < 0.001; ****P < 0.0001). Each dot represents an individual mouse

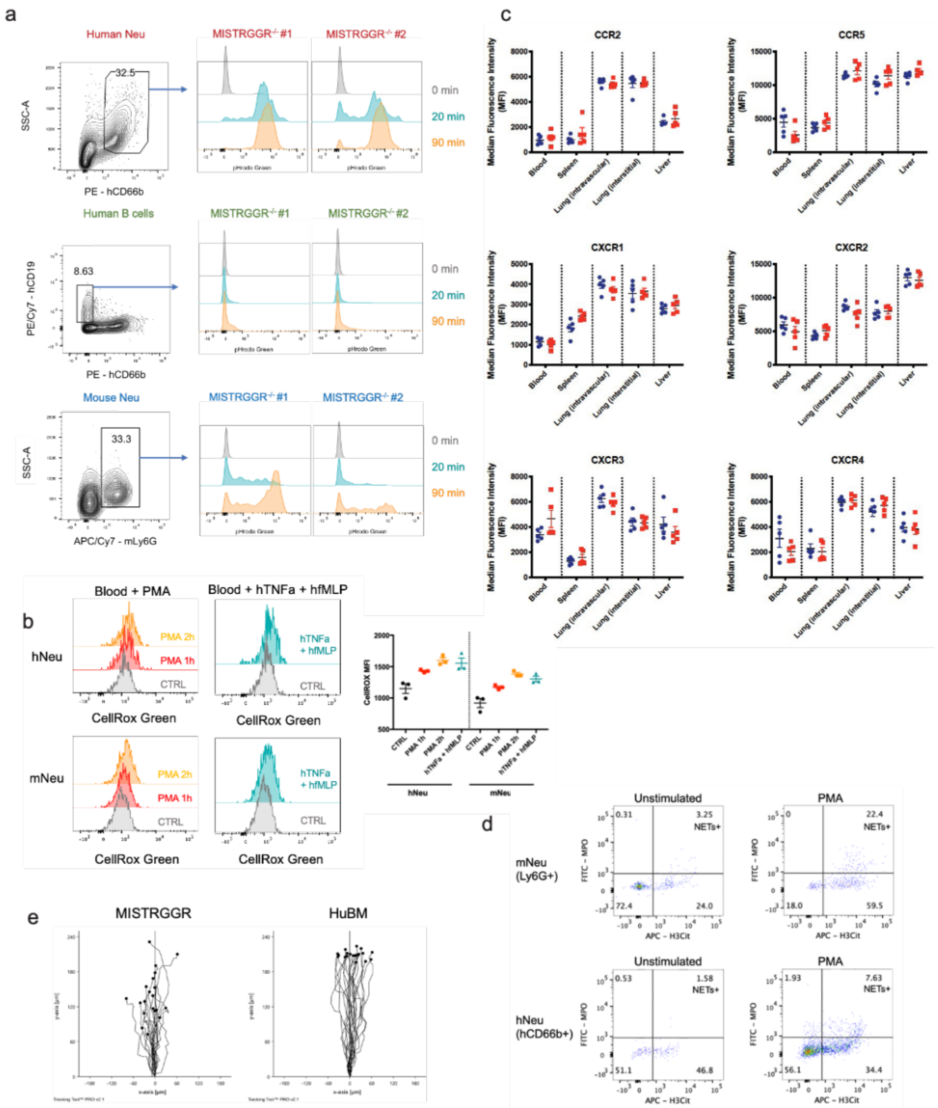

Fig. S3. Functionalities of reconstituted human neutrophils in MISTRGGR.

a. Representative flow cytometry analysis of phagocytosis by reconstituted human neutrophils (top), human B cells (middle) and mouse neutrophils (bottom) from the blood of engrafted MISTRGGR$^{-/-}$ mice. Blood samples were incubated with pHrodo™ Green E. coli BioParticles® Conjugate (10ug per 50ul blood) for the indicated times (0, 20 and 90 min) and fluorescent signals were analyzed by flow cytometry. Data were representative of at least 2 independent experiments.

b. Blood from MISTRGGR were stimulated with PMA (10 ng/ml) for 1 or 2hrs at 37C, or by hTNFa (20ng/ml) for 30 min, and stimulation with human fMLP (10uM) for 30 min at 37C. ROS production as measured by CellROX was analyzed by flow cytometry.

c. Chemokine receptor expression in hNeu (hCD66b+SSChi) from blood, spleen, liver (intravascular and interstitial) and liver at steady state.

d. Representative flow cytometry plots of NETs-forming neutrophils (MPO+ H3Cit+).Isolated human and mouse neutrophils from engrafted bone marrow were cultured unstimulated or with 20nM PMA for 4 hr at 37C. MPO and histone H3 were detected by flow cytometry.

e. Cell traces of human neutrophils from bone marrow of engrafted MISTRGGR$^{-/-}$ mice (left: MISTRGGR) or human neutrophils from healthy human bone marrow (right: HuBM) on X-Y plane migrating towards IL-8(1um) ex vivo using the EZ-taxiscan chamber.

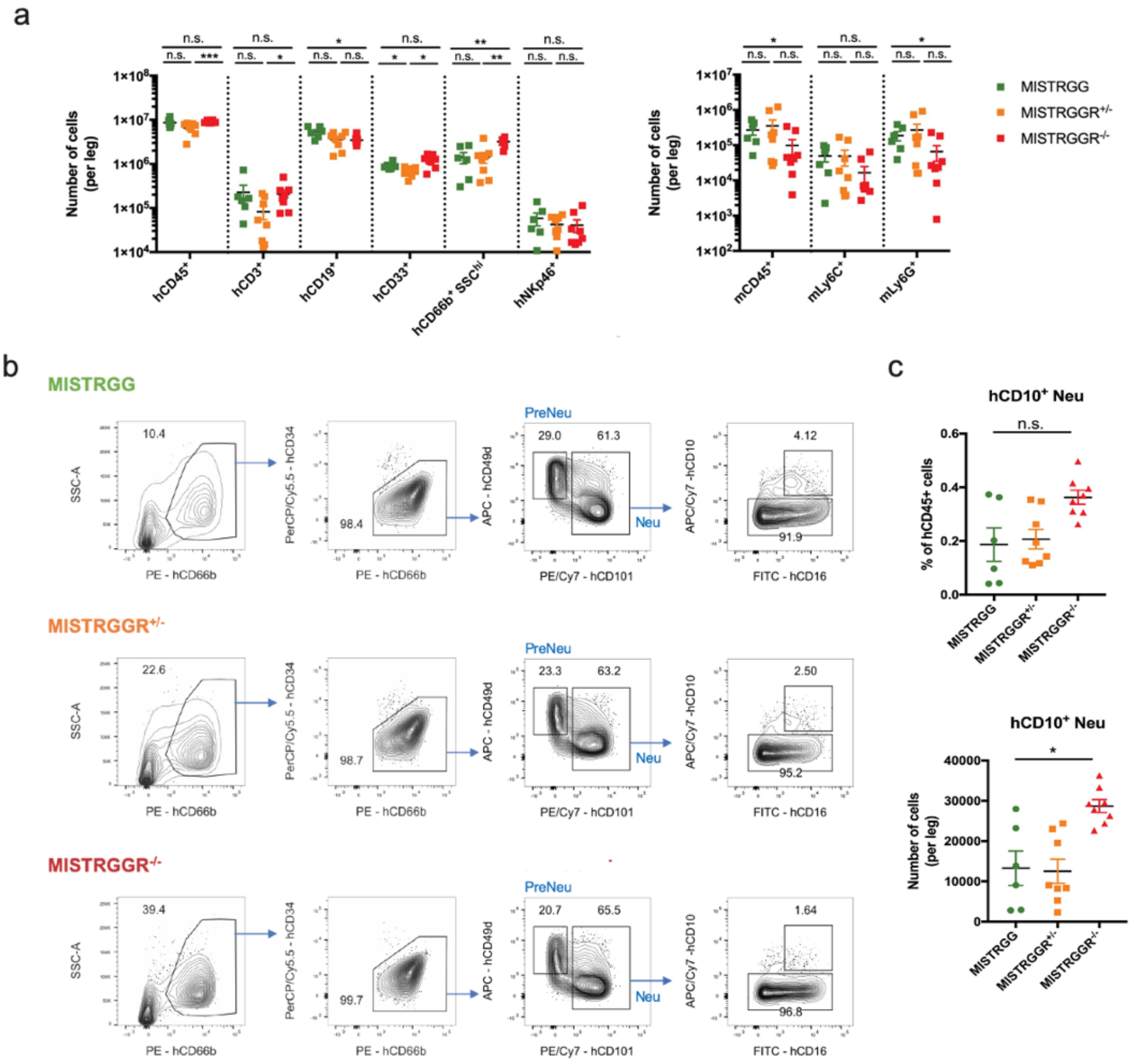

Fig. S4. Characterization of bone marrow engraftment in MISTRGG, MISTRGGR+/- and MISTRGGR$^{-/-}$ mice.

a. Quantifications of human (left) and mouse (right) immune lineages in engrafted bone marrow at week 8 post-engraftment (MISTRGG, n=6; MISTRGGR+/-, n=8; MISTRGGR-/-, n=8 mice).

b. Representative flow cytometry gating of total human neutrophils, Pre-Neu, and Neu.

c. Frequencies (left) and numbers (right) of CD10+ Neu (gated on hCD66b+ 15+ 34- SSChi) (MISTRGG, n=6; MISTRGGR+/-, n=8; MISTRGGR-/-, n=8 mice).

a-c. Data pooled from at least 2 independent experiments. All mice were irradiated with 150 Rads and intra-hepatically injected with 20,000 human fetal liver CD34+ cells at 1-3 days after birth. Data are shown as mean ± s.e.m.. P values determined by two-tailed Mann–Whitney test (*P < 0.05; **P < 0.01; ***P < 0.001; ****P < 0.0001). Each dot represents an individual mouse.

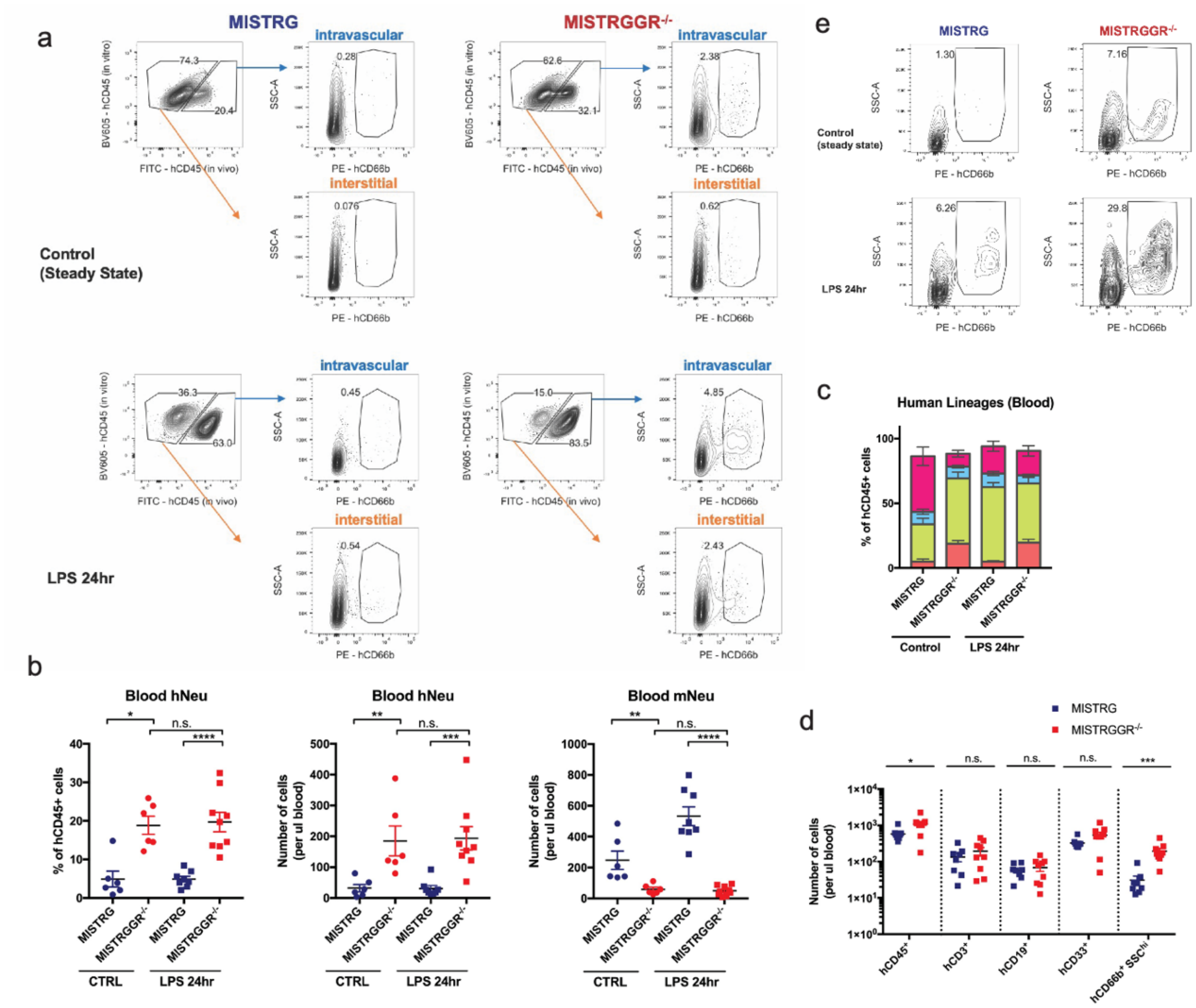

Fig. S5. Characterization of human immune cells in the blood of engrafted MISTRG and MISTRGGR$^{-/-}$ mice in response to LPS nebulization.

a. Representative flow plots of intravascular and interstitial human neutrophils (hCD66b+ SSChi) in engrafted lung.

b. Quantifications of human and mouse neutrophils in the blood at steady state and 24hr after LPS nebulization (CTRL MISTRG: n=6; CTRL MISTRGGR$^{-/-}$, n=6; LPS MISTRG, n=8; LPS MISTRGGR$^{-/-}$, n=9 mice).

c. Frequencies of human lineages in the blood at steady state and 24hr after LPS nebulization (CTRL MISTRG: n=6; CTRL MISTRGGR$^{-/-}$, n=6; LPS MISTRG, n=8; LPS MISTRGGR$^{-/-}$, n=9 mice).

d. Numbers of human lineages in the blood at 24hr after LPS nebulization (MISTRG, n=8; MISTRGGR$^{-/-}$, n=9 mice).

b-d, Data pooled from at least 2 independent experiments. All mice were irradiated with 150 Rads and intra-hepatically injected with 20,000 human fetal liver CD34+ cells at 1-3 days after birth. Data are shown as mean ± s.e.m.. P values determined by two-tailed Mann–Whitney test (*P < 0.05; **P < 0.01; ***P < 0.001; ****P < 0.0001). Each dot represents an individual mouse.

e. Representative flow plots of human neutrophils (hCD66b+ SSChi) infiltrated to BAL.

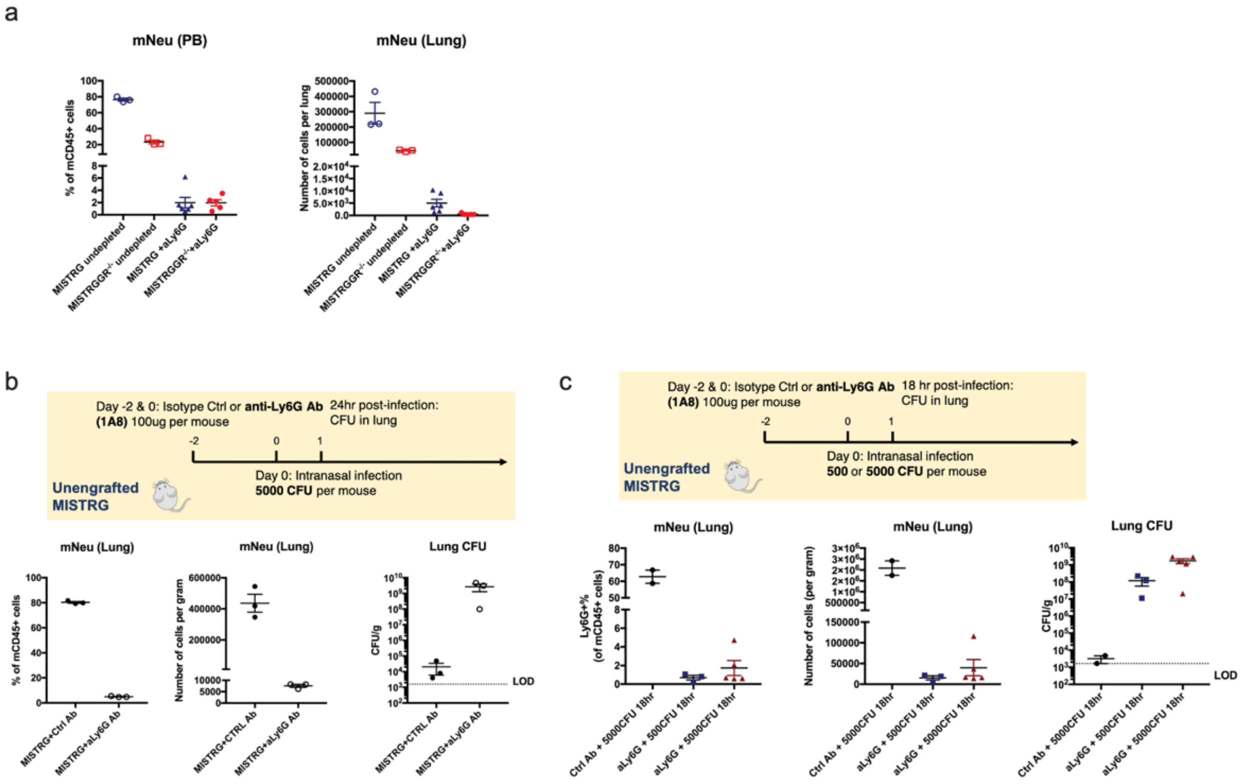

Fig. S6. Depletion of murine neutrophils with anti-Ly6G antibody.

a. Validation of murine neutrophil depletion after anti-Ly6G (1A8) antibody treatment. Blood and lung were analyzed at 18 hr post-infection (500 CFU) with untreated engrafted mice as control (MISTRG untreated, n=3; MISTRGGR$^{-/-}$ untreated, n=3; MISTRG +aLy6G Ab, n=6; MISTRGGR$^{-/-}$ +aLy6G Ab, n=5 mice). Left: Frequencies of mouse neutrophils (mCD45+ Ly6Cmid SSChi) in the blood; Right: Numbers of mouse neutrophils (mCD45+ Ly6Cmid SSChi) in the lung. Data pooled from 2 independent experiments.

b, c. Non-engrafted MISTRG mice were pre-treated with isotype control and anti-Ly6G (1A8) (2 doses of 100 µg, i.v.) for depletion of murine neutrophils, and then intranasally infected with P. aeruginosa. Frequencies and numbers of murine neutrophils and bacterial CFUs in the lung were quantified. b, Mice were infected with 5000 CFU and analyzed at 24 hr post-infection (n=3 mice).

c. Mice were infected with 500 or 5000 CFU of P. aeruginosa and analyzed at 18 hr post-infection (CTRL Ab, n=2; aLy6G Ab 500 CFU, n=3; 5000 CFU, n=5 mice). Data are shown as mean ± s.e.m.. Each dot represents an individual mouse.

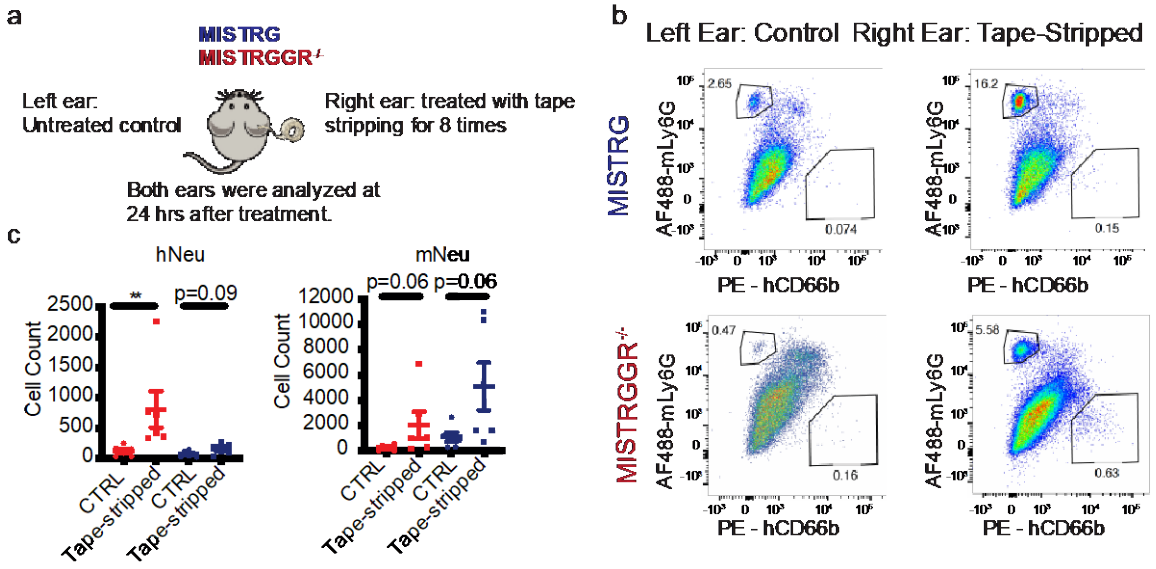

Fig. S7. Human neutrophils in MISTRGGR$^{-/-}$ mice respond to skin inflammation.
a. Schematic design of the tape-stripping experiment. Left ears of the mice were treated with tape transparent IV dressing (Tegaderm) stripping for 8 times. Both ears were collected for analysis of immune composition by flow cytometry at 24hrs post treatment.
b. Representative flow cytometry plots of human and mouse neutrophils in the ears.
c. Number of human and mouse neutrophils in left and right ears of tape-stripped MISTRG and MISTRGGR$^{-/-}$ mice (MISTRG, n=6; MISTRGGR$^{-/-}$, n=6 mice). Data pooled from at least 3 independent experiments. Data are shown as mean ± s.e.m. P values determined by two-tailed Mann–Whitney test (*P < 0.05; **P < 0.01; ***P < 0.001; ****P < 0.0001). Each dot represents an individual mouse.

Movies S1-2 (separate file). Human neutrophils from bone marrow of reconstituted MISTRGGR$^{-/-}$ mice chemotaxing towards IL8. The EZ-TAXIScan (ECI Frontier, MIC-1000) was used to investigate chemotaxis of human neutrophils. Related to figure 3e.
Movies S3-4 (separate file). Human neutrophils from fresh human bone marrow chemotaxing towards IL8. The EZ-TAXIScan (ECI Frontier, MIC-1000) was used to investigate chemotaxis of human neutrophils. Related to figure 3e.
